# The Redox-Sensing Regulator Rex Contributes to the Virulence and Oxidative Stress Response of *Streptococcus suis* Serotype 2

**DOI:** 10.3389/fcimb.2018.00317

**Published:** 2018-09-18

**Authors:** Haodan Zhu, Yong Wang, Yanxiu Ni, Junming Zhou, Lixiao Han, Zhengyu Yu, Aihua Mao, Dandan Wang, Hongjie Fan, Kongwang He

**Affiliations:** ^1^Institute of Veterinary Medicine, Jiangsu Academy of Agricultural Sciences, Nanjing, China; ^2^Jiangsu Co-Innovation Center for the Prevention and Control of Important Animal Infectious Disease and Zoonose, Yangzhou University, Yangzhou, China; ^3^College of Veterinary Medicine, Nanjing Agricultural University, Nanjing, China; ^4^Key Lab of Food Quality and Safety of Jiangsu Province, State Key Laboratory Breeding Base, Nanjing, China

**Keywords:** *Streptococcus suis*, redox-sensing regulator Rex, virulence, oxidative stress, electrophoretic mobility shift assays

## Abstract

*Streptococcus suis* serotype 2 (SS2) is an important zoonotic pathogen responsible for septicemia and meningitis. The redox-sensing regulator Rex has been reported to play critical roles in the metabolism regulation, oxidative stress response, and virulence of various pathogens. In this study, we identified and characterized a Rex ortholog in the SS2 virulent strain SS2-1 that is involved in bacterial pathogenicity and stress environment susceptibility. Our data show that the Rex-knockout mutant strain Δrex exhibited impaired growth in medium with hydrogen peroxide or a low pH compared with the wildtype strain SS2-1 and the complementary strain CΔrex. In addition, Δrex showed a decreased level of survival in whole blood and in RAW264.7 macrophages. Further analyses revealed that Rex deficiency significantly attenuated bacterial virulence in an animal model. A comparative proteome analysis found that the expression levels of several proteins involved in virulence and oxidative stress were significantly different in Δrex compared with SS2-1. Electrophoretic mobility shift assays revealed that recombinant Rex specifically bound to the promoters of target genes in a manner that was modulated by NADH and NAD^+^. Taken together, our data suggest that Rex plays critical roles in the virulence and oxidative stress response of SS2.

## Introduction

*Streptococcus suis* is an important zoonotic pathogen that has caused severe economic losses in the swine industry and endangered public health worldwide (Lun et al., 2007). Among the 33 serotypes defined based on capsular polysaccharide (CPS), serotype 2 (SS2) is the most virulent and the most frequently isolated in association with diseases in the majority of countries (Hill et al., 2005). The first human case of *S. suis* infection was reported in Denmark in 1968; by 2014, the total number of *S. suis* infections in humans was over 1600 (Goyette-Desjardins et al., 2014). In China, two large outbreaks of SS2 occurred in 1998 and 2005, resulting in 25 human cases with 14 deaths and 215 human cases with 38 deaths, respectively (Tang et al., 2006; Yu et al., 2006). During the past decades, numerous studies on *S. suis* have been performed; however, the pathogenesis of *S. suis* infection is still not entirely known (Segura et al., 2017).

During the infection process, pathogens encounter changing environments and host immune systems (Richardson et al., 2015). To deal with these hostile environments, pathogenic bacteria have evolved or acquired regulatory networks to sense and respond to environmental signals by modulating the expression of related genes. In *S. suis*, the signal regulatory systems contained within the two-component systems (TCSs) such as *SalK/R* (Li et al., 2008), *NisKR* (Xu et al., 2014), *CiaRH* (Li et al., 2011), and *SsSTK/SsSTP* (Zhu et al., 2011, 2014; Fang et al., 2017; Zhang et al., 2017), stand-alone regulators (SARs) such as *CcpA* (Willenborg et al., 2011, 2014) and *Rgg* (Zheng et al., 2011), and other regulators such as *CodY* (Feng et al., 2016), *PerR* (Zhang et al., 2012), *AdcR*, and *Fur* (Aranda et al., 2010) have been shown to be involved in bacterial metabolism and virulence. To gain further insight into the global regulatory networks of SS2, the role of other uncharacterized regulators should be investigated.

The redox-sensing regulator Rex was first discovered in *Streptomyces coelicolor* and is widely distributed among Gram-positive bacteria (Richardson et al., 2015). The crystal structures of Rex proteins from *Thermus aquaticus* and *Bacillus subtilis* in complex with NADH, NAD^+^, and/or DNA operator have been determined (Mclaughlin et al., 2010; Wang et al., 2011). Rex is composed of two domains, an N-terminal winged-helix DNA-binding domain and a C-terminal Rossmann-like domain involved in NADH binding and subunit dimerization. The DNA-binding activity of Rex proteins is modulated by the ratio of NADH to NAD^+^ concentrations (Brekasis and Paget, 2003; Mclaughlin et al., 2010). When the NADH/NAD^+^ ratio is low, Rex binds to target genes and represses the transcription of genes involved in NAD^+^ regeneration. In contrast, a high NADH/NAD^+^ ratio inhibits the DNA-binding activity of Rex and regulates the transcription of its target genes (Brekasis and Paget, 2003; Gyan et al., 2006; Pagels et al., 2010). The relationship between pathogenesis and the maintenance of an appropriate balance of reduced and oxidized NAD/NADH is not yet clear, but in some bacteria, such as *Staphylococcus aureus* (Pagels et al., 2010), *Enterococcus faecalis* (Vesić and Kristich, 2013), and *Streptococcus mutans* (Bitoun et al., 2012), the metabolic pathways under Rex control are implicated in virulence. In *S. aureus*, Rex is intimately involved in the survival of cells exposed to nitric oxide (NO), and it regulates genes involved in anaerobic respiration and fermentation (Pagels et al., 2010). The Rex ortholog in *S. mutans* contributes to the oxidative stress response and biofilm formation of bacteria (Bitoun et al., 2012; Bitoun and Wen, 2016). In *E. faecalis*, Rex has been shown to influence hydrogen peroxide (H_2_O_2_) accumulation (Vesić and Kristich, 2013).

Here, we characterized a Rex ortholog in *S. suis* (designed as SsRex) and examined the roles of SsRex in the oxidative stress tolerance and virulence of SS2. The isogenic mutant Δ*rex* strain exhibited increased susceptibility to oxidative stress agents, decreased survival in blood and macrophages, and attenuated virulence in murine infection models, suggesting that SsRex plays important roles in the pathogenicity of *S. suis*.

## Materials and methods

### Bacterial strains, plasmids, and growth conditions

The bacterial strains and plasmids used in this study are listed in Table 1. SS2 strain SS2-1 was isolated from a pig with a fatal case of septicemia in Jiangsu province in 1998 and has been confirmed as virulent by animal experiments (Zhu et al., 2014). SS2 strains were grown in Todd-Hewitt broth (THB) medium (Difco Laboratories) or plated on THB agar (THA) at 37°C. *Escherichia coli* strains were cultured in Luria-Bertani broth liquid medium or plated on Luria-Bertani agar. SS2 strains were grown in THB supplemented with 2% yeast extract (THY) for the preparation of competent cells. Culture media was supplemented with antibiotics (Sigma) as required at the following concentrations: spectinomycin (Spc), 100 μg/ml for SS2 and 50 μg/ml for *E. coli;* chloramphenicol (Cm), 4 μg/ml for SS2 and 8 μg/ml for *E. coli*.

**Table 1:** Bacterial strains and plasmids used in this study.

**Strains and plasmids**	**Characteristics or function^a^**	**Source or reference**
**SS2 STRAINS**		
SS2-1	Serotype 2, clinical isolated virulent strain, MRP^+^EF^+^SLY*^+^*	Our laboratory
*Δrex*	The Rex-deficient mutant with a background of SS2-1, Cm ^R^	This study
*CΔrex*	Complemented strain of *Δrex* ,Spc ^R^, Cm^R^	This study
***E. coli***		
DH5α	Cloning host for recombinant plasmid	Vazyme
BL21(DE3)	Host for overexpressed recombinant Rex protein	Vazyme
**PLASMIDS**		
pMD18 T	Clone vector	Takara
pET28a	Expression vector; Kan ^R^	Novagen
pET28a-Rex	pET-28a containing Rex, cloned from SS2-1 genome	This study
pSET4s	Temperature-sensitive *E. coli-S. suis* shuttle vector ,Spc^R^	Takamatsu et al., 2001b
pSET4sΔRex	Derived from pSET4s for deleting Rex in SS2-1	This study
pSET2	*E. coli- S. suis* shuttle vector*;* Spc^R^	Takamatsu et al., 2001a
pSET2-Rex	pSET2 containing the intact Rex and promoter; Spc^R^	This study

a *MRP, Muramidase released protein; EF, Extracellular factor; SLY, Suilysin; Cm^R^, chloramphenicol resistance; Spc^R^, spectinomycin resistant; Kan^R^, kanamycin resistant*.

### Gene inactivation and functional complementation

The thermosensitive suicide vector pSET4s was used for gene replacement in *S. suis* through homologous recombination (Takamatsu et al., 2001b). All the primers and plasmids used in this study are listed in Table 2. Two pairs of specific primers (L1/L2 and R1/R2) were used for cloning the Rex upstream and downstream homologous regions carrying *Hind*III/*Sal*I and *BamH*I/*EcoR*I restriction enzyme sites, respectively. DNA fragments were digested with the corresponding restriction enzymes and directionally cloned into vector pSET4s. The *cat* gene expression cassette was amplified from pBR326 with primers CAT1/CAT2 and inserted at the *Sal*I/*BamH*I sites to obtain the knockout vector pSET4sΔRex. To obtain the isogenic mutant Δ*rex*, SS2-1 competent cells were electrotransformed with pSET4sΔRex as described previously (Takamatsu et al., 2001b). SS2-1 containing pSET4sΔRex were grown at 28°C in the presence of Cm and Spc. Cells in mid-log growth phase were diluted 1,000-fold with THY broth containing Cm and cultured at 28°C to the early logarithmic growth phase. The culture was next moved to 37°C and incubated for 4 h before subsequent dilution and plating on THY-Cm plates and non-selective plates, which were then incubated at 37°C. Cultures were screened for vector-loss mutants that had exchanged their wild-type (WT) allele for a genetic segment containing the *rex* gene after homologous recombination via a double crossover. For all Cm^R^ transformants, colony PCR assays with primers IN1/IN2 were performed to detect the presence of *rex* in the genome. Candidate mutants in which the *rex* failed to be amplified were further verified by PCR assays with primers CAT1/CAT2, OUT1/CAT2, and CAT1/OUT2 and then confirmed by DNA sequencing.

**Table 2:** Primers used in this study.

**Primers name**	**Primer sequences (5′-3′)^a^**	**Functions**
L1	AAGCTTAACTAGATTTGGCAGGTATT	Upstream region of *rex*
L2	GTCGACTTTAGACTCCTTTATTTCGATA	
R1	GGATCCGCAATGAAAAAACCAGTTAT	Downstream region of *rex*
R2	GAATTCCATCGTTCATAACCACCC	
CAT1	GTCGACCACCGAACTAGAGCTTGATG	Cm^R^ expression cassette
CAT2	GGATCCTAATTCGATGGGTTCCGAGG	
Spc1	TAATAACGTAACGTGACTGG '	Spc^R^ gene cassette
Spc2	GGAGAAGATTCAGCCACT	
IN1	TGGTGCTGGTTACATCGG	An internal fragment of *rex*
IN2	CGCCAGTAGCGTTGTCGT	
OUT1	ATTGCTGCTATGGCGACTGC	For PCR screening
OUT2	TACGCAGTAAAATGTATGGCAAC	
CPS2J1	GTTCTTCAGATTCATCAACGGAT	For PCR screening
CPS2J2	TATAAAGTTTGCAACAAG GGCTA	
Rex-F	*GGATCC*GTGAAAAACGATAAAAAATCTGATA	*rex* coding sequence
Rex-R	*CTCGAG*TTATCCTTTTCGCATAAAGTAGAGT	
C1	*GCATGC*TTGACCGTGACATCTTTACAAA	*rex* open reading frame and its
C2	*GGATCC*TTGCTTATCCTTTTCGCATAA	promoter sequence
q-*rex*-F	ATATGGGGCGGGCCTTACTA	For qRT-PCR assays
q-*rex*-R	CACTTCTTGTGCCTTGACGC	
q-*aroA*-F	GAGAGTCGGTCATCGTAGAAAAAGA	For qRT-PCR assays
q-*aroA*-R	AATAAACTCCTGACCACCTTGGAT	
q-*dpr*-F	TGGTATATGCGTGGTCGTGG	For qRT-PCR assays
q-*dpr*-R	AAAGAGCAGCCAGATAGCGG	
q-*ilvE*-F	CCTTTATCTCCGTCCGCTCC	For qRT-PCR assays
q-*ilvE*-R	CGCGTAGTTACCACCAACCT	
q-*ldh*-F	CGTTGCCGGTGTGAAATTGT	For qRT-PCR assays
q-*ldh*-R	AAGTGCAACACCGATACCGT	
q-*pepT*-F	AGAATGTCCACTACCTACCAAAC	For qRT-PCR assays
q-*pepT*-R	GTATCCATGTGGGCGATGAA	
q-*purA*-F	GTTTGAAGGTGCGCAAGGAG	For qRT-PCR assays
q-*purA*-R	AGTCGGAAATGGTCCGTCAC	
q-*purB*-F	GATGAGGCTTGGGCTGAGTT	For qRT-PCR assays
q-*purB*-R	TGTCGTTGGCCTGCTTGTAT	
q-*treR*-F	AGGGCGTGGTTCAATGGT	For qRT-PCR assays
q-*treR*-R	CTCAAAGCCTGTCAAGTGCG	
q-a*dhE*-F	TGAAGGTCGTGCAGAAGGTC	For qRT-PCR assays
q-*adhE*-R	TGCGTCCGTAAGAACCACAA	
q-*arcA*-F	CACGGAACCGTGAAACCTTG	For qRT-PCR assays
q-*arcA*-R	TTGAAGCCAACATGCCGTTC	
q-*adhP*-F	TGCTGGCGTTACTTGCTACA	For qRT-PCR assays
q-*adhP*-R	GCACCAACTTCTTTGGCGAG	
q-*frdA*-F	TACAGAAGCAGTTCGTGGCG	For qRT-PCR assays
q-*frdA*-R	TGCAAGAGTAGCACCCGTTT	
q-*clpL*-F	AGGCTGGGACTCAGTATCGT	For qRT-PCR assays
q-*clpL*-R	CCCACGGGATAAAGCTGGTT	
EMSA-*rex*- F	AATTCACTATCTTGACGCTTAC	*rex* promoter amplification
EMSA-*rex-* R	CACTTTAGACTCCTTTATTTCG	
EMSA-*dpr*-F	AGCCATAGGGCTACGAC	*dpr* promoter amplification
EMSA-*dpr*-R	CTTGATTCAAAACAGCCT	
EMSA-*ldh*-F	TCGCACTATCTCCATGCG	*ldh* promoter amplification
EMSA-*ldh*-R	CCGTCACCGACAAGGATT	
EMSA-*ilvE*-F	TACTGCCCAACTCATCAC	*ilvE* promoter amplification
EMSA-*ilvE*-R	CAAGAGCAAACGGTTAACT	
EMSA-*treR*-F	TCTTTTGATACGCCTTCG	*treR* promoter amplification
EMSA- *treR*-R	TGTTCTGTCGGTAGGGAG	
EMSA-*adhE*-F	CTGCTTTCATAAGCAGACC	*adhE* promoter amplification
EMSA-*adhE*-R	CAATTTTCTTTCGGGGA	
EMSA-*acrA*-F	CTTGGCTCTTCTTTATTGG	*acrA* promoter amplification
EMSA-*acrA*-R	GTACGCTTTCCTGCCTCT	
EMSA-*clpL*-F	TCAGGACTTGCAGGACACT	*clpL* promoter amplification
EMSA-*clpL*-R	ATGTTGCCCATGAGTTGA	
EMSA-*frdA*-F	GATAGACGAAGTGGAGCA	*frdA* promoter amplification
EMSA-*frdA*-R	GACTTACCTCCACACGC	
EMSA-*impdh*-F	ATGGAGGCAGGACAGGTAT	*impdh* promoter amplification
EMSA-i*mpdh*-R	GTTCTTTCCTTTCTTTTGGG	
*16SrRNA*-F	GCATAACAGTATTTACCGCATGGTAGAT	EMSA negative control
*16SrRNA*-R	TTCTGGTAAGATACCGTCAAGTGAGAA	

a *Underline nucleotides denote enzyme restriction sites*.

The SsRex promoter sequence was predicted based on computer analysis by using BPROM (http://www.softberry.com/berry.phtml). For functional complementation, the promoter region (372 bp) and open reading frame of the *Rex* sequence (639 bp) were amplified by PCR using specific primers C1/C2 (carrying *Sph*I/*Eco*RI restriction enzyme sites) and then cloned into pSET2 (Takamatsu et al., 2001a) to obtain the recombinant plasmid pSET2-Rex, which was subsequently electroporated into Δ*rex*. The complemented strain CΔ*rex* was screened on THY plates with double selection pressure of Spc and Cm and further confirmed by PCR assays using primers IN1/IN2, CAT1/CAT2, and Spc1/Spc2.

### Adherence and invasion assays

The adherence and invasion assays were performed using HEp-2 cells as described previously, with some modifications (Feng et al., 2016). HEp-2 cells were cultured in DMEM medium (Hyclone), maintained at 37°C, and supplemented with 10% (vol/vol) fetal bovine serum (FBS) (Sijiqing, Hangzhou, China). In the adherence assays, log-phase bacteria (10^7^ CFU) were added to the wells of a 24-well tissue culture plate containing a monolayer (10^5^ cells) of HEp-2 cells (multiplicity of infection [MOI] of 100 bacteria/HEp-2 cell). The plates were incubated for 2 h at 37°C in 5% CO_2_ to allow cell adherence by the bacteria. The monolayers were then washed four times with PBS, digested with 100 μl of trypsin for 15 min at 37°C, and then disrupted by the addition of 900 μl of sterile deionized water on ice followed by repeated pipetting to release all bacteria. Aliquots (100 μl) diluted 10^2^ to 10^4^ in PBS were used for quantitative plating.

For the invasion assays, log-phase SS2 (10^7^ CFU) at a MOI of 100 were incubated with HEp-2 cells at 37°C for 2 h, and then the culture suspensions were removed. DMEM culture medium containing ampicillin (100 μg/ml) was added into the wells of cells infected with SS2 for 2 h to kill the bacteria outside the host cells. The cells were then washed four times with PBS. A 100-μl sample of the last PBS wash solution was plated on THA to test whether 100% of extracellular bacteria were killed after the antibiotic treatment. The cells were treated with 100 μl of trypsin for 15 min and then disrupted by the addition of 900 μl of sterile deionized water on ice. Aliquots (100 μl) of serial dilutions in PBS were used for quantitative plating. The experiments also included negative control cells without SS2 infection, which were plated on THA. The adherence and invasion assays were each performed in triplicate and repeated three times.

### Acid and oxidative stress challenges

To evaluate the role of SsRex in acid and oxidative stress, SS2 WT and its mutant strains were challenged with HCl and H_2_O_2_ according to previously described protocols (Han et al., 2012; Li et al., 2014). In the acid tolerance assays, log-phase bacteria cultured in THB (pH 7.0) were harvested by centrifugation at 3000 ×*g* at 4°C for 10 min, washed once with 0.1 M glycine buffer (pH 7.0), and then resuspended using THB with various pH values (4.0, 5.0, 6.0, and 7.0), which were achieved by adjustment with HCl. The suspensions were incubated for up to 4 h at 37°C, and the numbers of surviving cells were determined by plating them on THA plates in triplicate, followed by incubation at 37°C for 24 h (Han et al., 2012).

In the oxidative stress tolerance assays, log-phase bacteria were pelleted, washed, and resuspended in 0.1 M glycine buffer, pH 7.0. H_2_O_2_ was added to the cell suspension to create a final concentration of 10 mM (Han et al., 2012), and the cells were incubated for 15 min, 30 min, or 45 min. The catalase was then added immediately (5 mg/ml; Sigma) to the samples to inactive the H_2_O_2_ (Li et al., 2014). Lastly, the survival rate was calculated by plating the samples in triplicate on THA plates. These experiments were performed in triplicate and repeated three times.

### Intracellular survival assays

Murine macrophage RAW264.7 cells were cultured in DMEM supplemented with 10% FBS in 24-well tissue culture plates at a concentration of 4 × 10^5^ cells/well for intracellular survival assays as described previously, with some modifications (Tang et al., 2012). Log-phase bacteria were pelleted, washed twice with sterile PBS, and resuspended in fresh serum-free DMEM without antibiotics. The RAW264.7 cells were infected at a MOI of 10. After being co-cultured with the SS2 strains for 1 h at 37°C in 5% CO_2_, the macrophages were washed four times with PBS and then incubated in DMEM with 1% FBS containing ampicillin (100 μg/ml) for the duration of the assays. For measuring the survival of intracellular bacteria, infected cells were sampled at 1 h after the addition of antibiotics (time 0) and again at 2, 4, 6, 8, and 10 h. The cells were washed three times with sterile PBS and then incubated with 0.02% Triton X-100 for 15 min at 37°C to lyse the macrophages and release intracellular bacteria. Serial dilutions of these lysates were plated on THA plates and incubated overnight at 37°C. Colonies were counted to determine the number of intracellular bacteria. To analyze the number of bacteria surviving over time compared with the initial number of intracellular bacteria, the relative CFU (rCFU) was calculated as follows: rCFU at time point x/CFU at time point 0 (Cumley et al., 2012). These experiments were performed with three biological replicates, each with two technical replicates.

### Survival in blood

The survival of SS2 in whole blood was determined as described in previous studies (Feng et al., 2016). Briefly, 50 μl of mid-log-phase cultures of the SS2-1 and Δ*rex* strains, which had been pelleted, washed twice with sterile PBS, and adjusted to an OD_600_ of 0.1, were inoculated into sterile Eppendorf tubes pre-filled with 450 μl of fresh heparinized pig blood from clinically healthy pigs. The blood-bacteria mixtures were incubated at 37°C for 2 h. Surviving bacteria were then diluted and plated on THA. These experiments were performed in duplicate and repeated three times.

### Mouse infections

All animal experiments were approved by the Science and Technology Agency of Jiangsu Province. Approval (ID: XYSK 2015-0020) was also granted by the Jiangsu Academy of Agricultural Sciences Experimental Animal ethics committee. All efforts were made to minimize the suffering of the animals. The BALB/c mice (female, 4–6 weeks old) were obtained from Yangzhou Laboratory Animal Research Center, Yangzhou, China. A total of 80 female BALB/c mice were randomly classified into 10 groups with 8 mice per group. Log-phase cultures of SS2 strains were centrifuged, and the resulting cell pellets were washed twice in PBS and then suspended in THY. For each strain, three groups of experimental mice were injected intraperitoneally (ip) with 1.0 ml of bacterial suspension at the following concentrations: 4.20 × 10^7^ CFU/ml, 8.40 × 10^6^ CFU/ml, and 1.68 × 10^6^ CFU/ml. The last group mice (8 control mice) were inoculated with only the medium solution (THY). Mortality was monitored until 7 days post-infection, after which the 50% lethal dose (LD_50_) value was calculated using the method of Reed and Muench (Reed Lj, 1938).

### Protein digestion and iTRAQ labeling

All the SS2 strains were grown in THB in triplicate (three WT strain SS2-1 and three mutant strain Δ*rex*).When the SS2 cultures reached an OD600 of 0.7, 80 ml of sample were taken from each culture and pooled (240 ml total volume) (Redding et al., 2006). The samples were centrifuged at 10,000 × *g* for 5 min at 4°C, and the resulting cell pellets were then washed twice with PBS (Shen et al., 2013). iTRAQ analysis was carried out at Wuhan GeneCreate Biological Engineering Co., Ltd. (Wuhan, GeneCreate, China). Protein digestion was performed as previously described (Jing et al., 2008) with some modifications. Briefly, bacterial cell pellets were ground to powder in liquid nitrogen and incubated in dissolution buffer (8 M urea/100 mM triethylammonium hydrogen carbonate buffer [TEAB], pH 8.0) containing 1 mM PMSF and 2 mM EDTA (final concentration) for 5 min, and 10 mM DTT (final concentration) was then added to the sample. The suspension was sonicated for 15 min and then centrifuged at 4°C at 14,000 ×*g* for 20 min. The resulting supernatant was mixed with four volumes of precooled acetone at −20°C overnight. After another centrifugation, the resulting protein pellets were air-dried and resuspended in 8 M urea/100 mM TEAB (pH 8.0). Protein samples were reduced with 10 mM DTT at 56°C for 30 min and then alkylated with 50 mM iodoacetamide (IAM) for 30 min in the dark. The protein concentration was measured using the Bradford Protein Assay Kit (Beyotime,Shanghai,China). After being diluted 5× with 100 mM TEAB, equal amount of proteins from each sample were used for tryptic digestion. Trypsin was added at an enzyme protein ratio of 1:50 (w/w), and the digest reaction was performed at 37°C for 12–16 h. After digestion, peptides were desalted using C18 columns, and the resulting desalted peptides were dried with a vacuum concentration meter. The dried peptide powder was later re-dissolved with 0.5 M TEAB and processed according to the manufacturer's instructions for the iTRAQ Reagent-8 plex Multiplex Kit (AB Sciex U.K. Limited). The peptides from the WT strain SS2-1 were labeled with iTRAQ tag 119, and the peptides from the mutant strain Δ*rex* were labeled with tag 121. The peptide samples were fractionated using a Durashell C18 column (5 μm, 100 Å, 4.6 × 250 mm) on an Ultimate 3000 HPLC system (Thermo DINOEX, USA) operating at 1 ml/min. Peptides were separated by increasing acetonitrile (ACN) concentrations under high pH (pH 10) conditions. Fractions were collected at 1-ml intervals and pooled into 12 fractions. Each fraction was dried with a vacuum concentration meter.

### LC-ESI-MS/MS analysis

The peptide samples were dissolved in 2% acetonitrile/0.1% formic acid and analyzed using a Triple TOF 5600+ mass spectrometer coupled with the Eksigent nanoLC System (SCIEX, USA) as previously described (Lin et al., 2015). The raw files collected from the Triple TOF 5600 were interpreted by ProteinPilot version 4.5 (July 2012, Applied Biosystems; Foster City, CA, USA). MS/MS spectra were searched against the Uniprot *S. suis* database (80,299 items, updated Jan 2017). The following search parameters were used: the instrument was set as TripleTOF 5600 plus with cysteine carbamidomethylation and 8 multiplex iTRAQ labeling was set as a fixed modification, methionine oxidation as a variable modification, and digestion by trypsin with at less one missed cleavage. The ratio of label 119 to label 121 represents the expression of proteins with the protein identification confidence of a 1% false discovery rate (Unwin et al., 2010). The proteins were considered overexpressed when the iTRAQ ratio was above 1.5 and under-expressed when the iTRAQ ratio was lower than 0.67 (Yu et al., 2018).

### Transcriptional analysis

Total RNA was extracted from *in vitro* late log-phase (OD_600_, 0.6–0.8) bacterial culture using the EZNA bacterial RNA kit (Omega, Beijing, China) according to the manufacturer's protocol. cDNA was reverse transcribed using a PrimeScript RT reagent kit with gDNA Eraser (TaKaRa, Dalian, China). The two-step relative quantitative real time PCR (qRT-PCR) was performed to analyze the expression profile of these selected genes using a SYBR Premix Ex Taq kit (TaKaRa). The ABI 7500 RT-PCR system was used for relative qRT-PCR. The gene-specific primers used for the qRT-PCR assays are listed in Table 2. The housekeeping gene *aroA* (encoding 5-enolpyruvylshikimate 3-phosphate synthase) used as the internal control was also amplified under the same conditions to normalize reactions. Reactions were performed in triplicate. A dissociation analysis of amplification products was performed at the end of each PCR assay to confirm that only one PCR product was amplified and detected. The relative fold change was calculated based on the 2^−ΔΔ*Ct*^ method (Livak and Schmittgen, 2001).

### Expression and purification of the recombinant protein SsREX

The whole open reading frame of SsRex was amplified by PCR with primers Rex-F and Rex-R containing added *BamH*I and *Xho*I recognition sites (listed in Table 2). The PCR product was restriction-enzyme digested with *Bam*HI and *Xho*I and then inserted into the similarly digested pET-28a vector (Novagen) to generate the recombinant plasmid pET28a-Rex, which was then transformed into *E. coli* BL21 (DE3) cells. The recombinant Rex protein was expressed via IPTG (isopropyl–D-thiogalactopyranoside) induction. The resulting fusion protein was purified using a HisTrap HP column (GE Healthcare, Shanghai, China) according to the manufacturer's guidelines. The expression and purity of the fusion protein were determined by sodium dodecyl sulfate-polyacrylamide gel electrophoresis (SDS-PAGE). The purified protein was stored at 4°C for later use in electrophoretic mobility shift assays.

### Electrophoretic mobility shift assays (EMSAs)

Protein–DNA interactions were analyzed by performing EMSAs according to previously described protocols with some modifications (Pagels et al., 2010; Bitoun et al., 2012). The promoter regions of the potential target genes, which were selected according to our comparative proteomics results and findings from previous studies in other pathogens, were amplified by PCR from SS2-1 gDNA using the specific primers shown in Table 2 and purified by using the High Pure PCR product purification kit (Axygen, Hangzhou, China). The purified PCR fragments were incubated with increasing amounts of rSsRex in reaction buffer containing 20 mM Tris pH 8.0, 1 mM EDTA, 75 mM KCl, 2 mM DTT, and 10% glycerol for 20 min at 30°C. When NAD^+^ or NADH was included for EMSAs, the samples were incubated for an additional 15 min (Pagels et al., 2010; Bitoun et al., 2012). Promoter fragments of *impdh* (Inosine 5-monophosphate dehydrogenase) (Zhou et al., 2014) lacking a putative Rex-binding site and 16sRNA were used as negative controls. Another two control reactions in which only included the DNA fragment or rRex protein were also performed. The reaction mixtures were separated on a 6% polyacrylamide gel in 0.5× Tris-borate-EDTA for 60 min. After electrophoresis, the DNA mobility shift was visualized by staining with ethidium bromide using a Tanon 2500R imager.

### Statistical analysis

All the statistical analyses were performed using GraphPad Prism 5. One-way analysis of variance (ANOVA) tests were used to analyze the oxidative stress, bacterial adherence, and survival in whole blood assays. Mann-Whitney tests were used to analyze the bacterial load in all organs examined. Two-way ANOVAs were performed on the qRT-PCR results. Values of *P* that were <0.05 were considered significant.

## Results

### Bioinformatics analysis of rex in the *S. suis* genome

The *rex* gene of SS2-1 has a 639-bp open reading frame and encodes a protein composed of 213 amino acids with a predicted molecular mass of 23.7 kDa and an isolectric point of 8.27. BlastN analysis using the *rex* sequence of SS2-1 confirmed the presence of *rex* in all 35 complete *S. suis* genomes available in the National Centre for Biotechnology Information database as of 31 Dec 2017 (data not shown). BlastP analysis revealed that SsRex (possessing N-terminal DNA-binding sites and a C-terminal Rossmann-fold NAD(P)H/NAD(P)(+)-binding [NADB] domain) exhibited more than 78% amino acid sequence identity with the Rex homolog proteins in other *Streptococci*. Multiple sequence alignments of the redox-sensing regulator Rex from *S. suis* and other *Streptococci* revealed that Rex is highly conserved among *Streptococcus* species. Protein homology modeling was performed to predict the structure of SsRex, which may be useful for studying its active sites and designing therapeutics against *S. suis* infection. The secondary structure of SsRex is predicted to consist of nine α-helices, eight β-sheets, and three coils (Data not shown).

**Figure 1:**
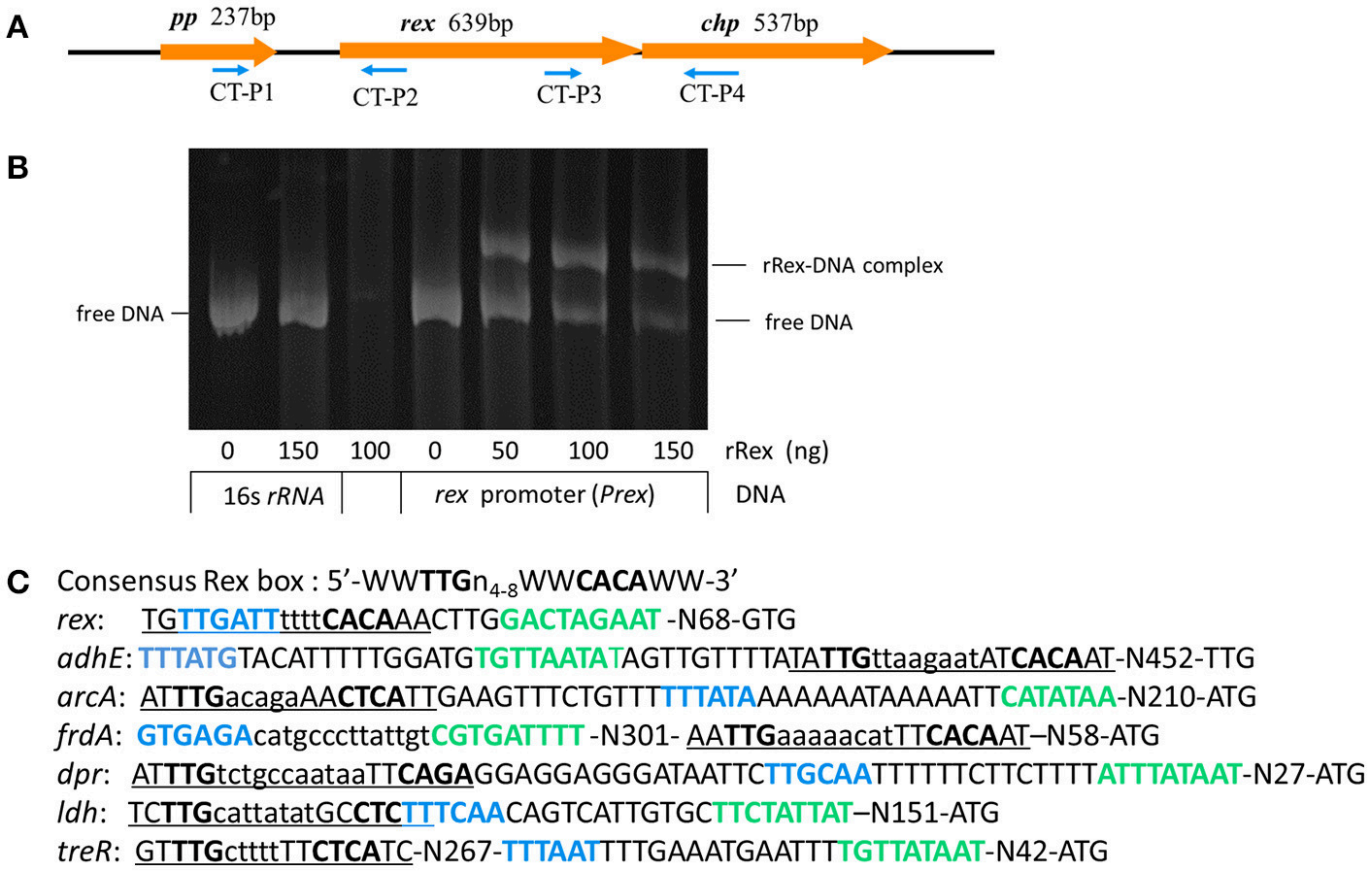
Identification and characterization of redox-sensing regulator Rex in SS2. **(A)** Schematic diagram of regions flanking *rex*, with the arrows indicating the direction of transcription and the numbers above indicating the sizes of the respective open reading frames in base pairs. **(B)** EMSA analysis shows interaction of *rex* promoter (*Prex*) with recombinant Rex (rRex) protein. Inclusion of rRex resulted in mobility shift and such interaction was concentration-dependent. **(C)** The promoter regions of selected genes identified in SS2-1 that contain putative Rex binding sites. The−35 regions (blue font) and−10 regions (green font) as determined by BPROM programs are shown in bold type and putative Rex-binding sites are underlined. W indicates A or T.

The *rex* gene of SS2-1 is flanked downstream by *chp*, which encodes a conserved hypothetical protein, and upstream by *pp*, which encodes a putative peptidase (Figure 1A). Computer based analysis using BPROM (www.softberry.com), a bacterial sigma70 promoter recognition program, identified a putative −10 and −35 element of the *rex* promoter situated 97 nucleotides upstream of the translational start site (GTG) of Rex. The transcriptional terminator signal was predicted by an online tool (http://rna.igmors.u-psud.fr/toolbox/arnold/). It identified an inverted repeat sequence (TGAATATAAAACAGGGGATCATACAG**AACCCCTG**CTTTCTTATACCCA) (underline nucleotides denote palindrome sequence) situated 36 nucleotides downstream of the stop coden (TAA) of *rex* that may be a transcriptional terminator, suggesting that *rex* is monocistronically transcribed. Reverse transcription-PCR (RT-PCR) was performed using the primers CT-P1/P2 and CT-P3/P4 to analyze the transcription of genes adjacent to *rex* and the results showed that *rex* was a single transcript unit, confirming the predicted results of online tools (Supplementary Figure 1). The binding sequence of Rex is highly conserved in Gram-positive bacteria. The reported consensus sequences in *S. coelicolor* (5′-TGTGAACNNNTTCACA-3′) (Brekasis and Paget, 2003), *B. subtilis* (5′ -WWTGTGAANTNNTNNNCAAW-3′; W represents either A or T) (Wang et al., 2008), *S. aureus* (Pagels et al., 2010) (5′ -TTGTGAAWWWWTTCACAA-3′), and *S. mutans* (TTGTGAANNNNTTCACAA) (Bitoun et al., 2012) are very similar. In the SS2-1 strain, a putative Rex-binding sequence (TGTTGATTTTTTCACAA) in the Rex promoter region, which overlapped the −35 element, provides a feasible mechanism by which Rex feedback autoregulation could occur (Figure 1C). The EMSA results show that recombinant Rex (rRex) was able to bind to the promoter regions of *rex*(*Prex*), and displayed a dose-dependent mobility shift to the higher molecular mass of rRex-*Prex* complexes. However, the rRex protein did not bind to the control DNA fragment from 16S*rRNA*, and no binding was observed under these conditions, even with 150 ng of rRex protein (Figure 1B). These results further suggest that SsRex is an authentic Rex transcriptional factor that is poised for autoregulation.

### Construction of *rex* knockout mutant and complemented strains

An isogenic *rex* mutant of SS2 strain SS2-1 was constructed through homologous recombination (Supplementary Figure 2A). One candidate mutant in which the *rex* gene failed to be amplified was found from more than 180 Cm^R^ transformants. The allelic replacement of the *rex* gene by the Cat^R^ in the mutant strain was confirmed by multiple PCR analysis (Supplementary Figure 2B), and DNA sequencing (data not shown). The complemented strain was screened on THY plates with double selection pressure of Spc and Cm and further confirmed by PCR analysis (Supplementary Figure 2C), and DNA sequencing (data not shown). A previous study reported that the bacterial cells carrying the pSET vectors should be grown under appropriate antibiotic pressures, especially when the *recA* mutant strains are used as the hosts (Takamatsu et al., 2001a). So we detected the expression levels of *rex* in SS2-1, Δ*rex*, and *C*Δ*rex* in presence or absence of antibiotic pressure by using qRT-PCR. The expression level value of *rex* in the WT SS2-1 was set as 1.0 and the results were shown as relative expression ratios compared to SS2-1. The qRT-PCR analysis showed that the expression level of *rex* in Δ*rex* was not detectable, and that of in CΔ*rex* with antibiotic, and CΔ*rex* in the absence of antibiotic pressure were decreased by 0.32, and 0.45, respectively, compared with the WT strain (Supplementary Figure 2D). There was no significant difference between the CΔ*rex* strain under the two conditions with or without antibiotic. The qRT-PCR results confirmed the *rex* was transcribed after complementation regardless of the antibiotic pressure.

### Δ*rex* demonstrates reduced tolerance to acid and oxidative stress agents

**Figure 2:**
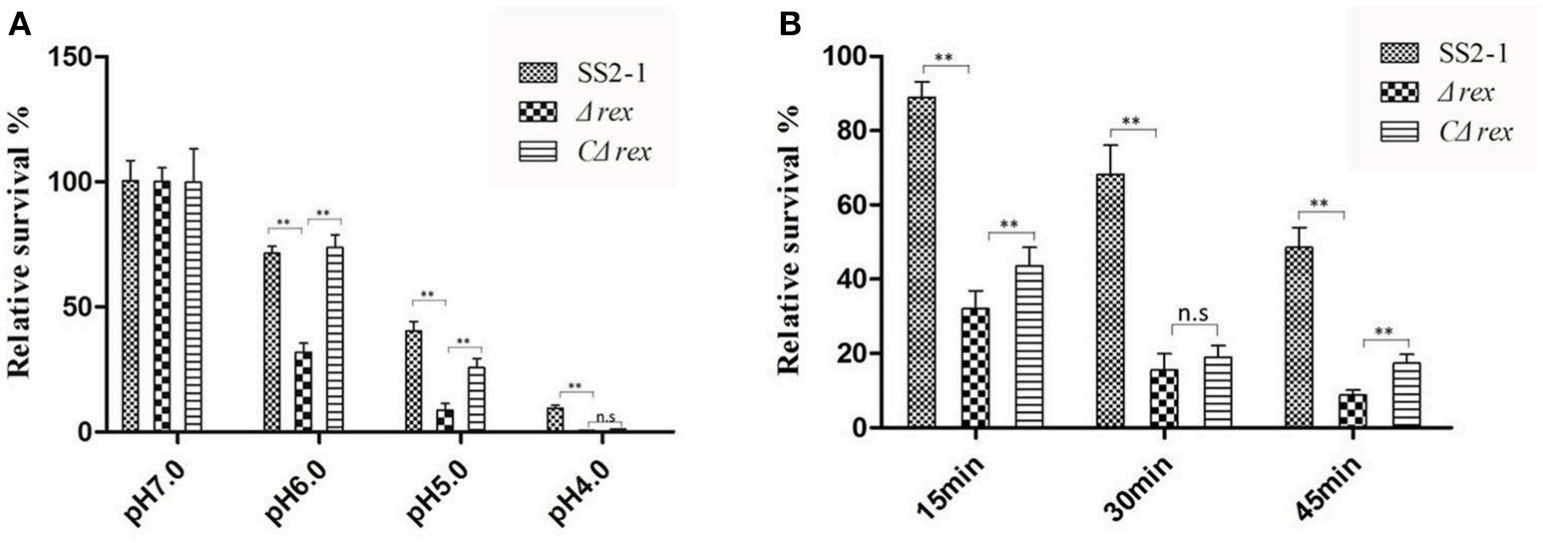
**(A)** In acid challenge assay, log phase SS2 cultures were harvested and washed once with 0.1 M glycine buffer (pH 7.0), and then resuspended using THB with various pH values (4.0, 5.0, 6.0, and 7.0), which were achieved by adjustment with HCl. The suspensions were incubated for up to 4 h at 37°C and the numbers of surviving cells were determined by plating them on THA plates in triplicate. **(B)** In H_2_O_2_ challenge assay, log phase SS2 were pelleted, washed and resuspended in 0.1 M glycine buffer (pH 7.0). H_2_O_2_ was added to the cell suspension to create a final concentration of 10 mM and incubation for 15, 30, and 45 min, respectively. Then catalase was added immediately (5 mg/mL; Sigma) to the samples to inactive H_2_O_2_. Surviving cells were diluted appropriately, plated on THA plates. The percentage of the CFU was normalized to WT group designed as 100% (n. s, *p* > 0.05, **p < 0.01).

During the infection process, *S. suis* is exposed to a hostile environment with various stress factors, including nutritional deprivation, increased osmolality, lowered pH, and reactive oxygen species (ROS) generated by host phagocytes (Zhu et al., 2014). Rex regulators play important roles in acid and oxidative stress tolerance in several pathogens (Pagels et al., 2010; Bitoun et al., 2012). To test whether SsRex affects the acid and oxidative tolerance of SS2, we compared the survival rates of SS2-1, Δ*rex*, and CΔ*rex* strains under these stress conditions *in vitro*. In a neutral pH environment, the survival rates of these three SS2 strains (WT SS2-1, Δ*rex*, and CΔ*rex*) were similar; in contrast, as the pH of the medium was lowered, the bacterial survival of Δ*rex* rapidly decreased compared with the WT and the CΔ*rex* strains (Figure 2A). The results show that Δ*rex* had reduced tolerance to acidic pHs (*p* < 0.01). In addition, when these three SS2 strains were challenged with H_2_O_2_ (10 mM H_2_O_2_ in THB), Δ*rex* was significantly more susceptible to H_2_O_2_ compared with the WT and CΔ*rex* strains. However, there was no significant difference between the H_2_O_2_ susceptibility of Δ*rex* and CΔ*rex* following a 30 min-incubation in the oxidative stress assay (Figure 2B). These results suggest that the expression of SsRex contributes to the resistance of SS2 to environmental stresses.

### SsRex did not affect the SS2 adherence to or invasion of HEp-2 cells

Successful establishment of infection by *S.suis* requires adhesion to host cells, colonization of tissues, and in certain cases, cellular invasion followed by intracellular multiplication, dissemination to other tissues (Tang et al., 2006). HEp-2 cells were used to evaluate the effcts of SsRex on the SS2 adhesion to and invasion of mammalian cells. Compared with the WT strain SS2-1, Δ*rex* showed similar adherence and invasion levels (*p* > 0.05) (Supplementary Figure 3). These results indicate that SsRex was not crucial for SS2 adherence to or invasion of HEp-2 cells.

### SsRex deficiency reduced Ss2 survival in whole blood and macrophages

**Figure 3:**
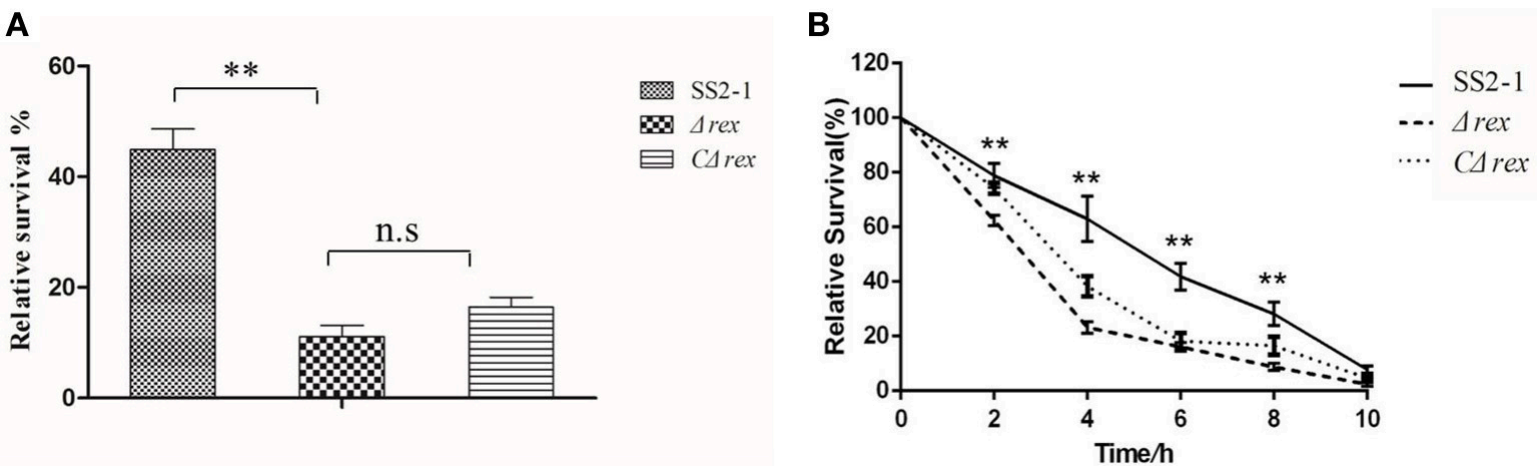
**(A)** Survival of WT SS2-1 in pig whole blood compared to that of the mutant strain Δ*rex* and complemented strain CΔ*rex*. Mixtures were incubated at 37°C for 2 h. A value of 100% was given to the CFU at time 0 h. The survival rate of Δ*rex* was significantly reduced compared to SS2-1. **(B)** Intracellular growth of SS2 in RAW264.7 macrophages. The macrophages cells were infected with mid-log growth phase SS2 strains at a MOI of 10 (bacteria: macrophage).The samples were taken 1 h after the addition of antibiotics (time zero) and then at 2, 4, 6, 8, and 10 h. The relative numbers of CFU (rCFU) were estimated by plating out the lysates of infected macrophages and counting the numbers of CFU at each time point. Asterisks indicate the time points when the intracellular bacteria survival rates elicited by the Δ*rex* were significantly lower than those produced by WT infection.

*S. suis* invasion from the mucosal surface into deeper tissues and its blood circulation are critical events in the development of disease. Therefore, *S. suis* survival in the blood is central to its pathogenesis (Fittipaldi et al., 2012). To determine whether the deletion of SsRex affected the survival of SS2 in whole blood, we measured the survival ability of SS2 strains in whole pig blood. The survival rate of the WT strain SS2-1 was 45.0%, which was significantly higher than that of Δ*rex*, which had a survival rate of 11.1% (*p* < 0.01) (Figure 3A). Compared with the mutant strain Δ*rex*, the survival rate of the complemented strain CΔ*rex* was increased up to 16.2%. However, although the entire *rex* gene is complemented in CΔ*rex* (Supplementary Figure 2D), its survival rate in the blood bacterial assay was not as high as that of the WT strain (Figure 3A).Similar finding had also been reported in other study (Ju et al., 2012).

Once *S. suis* reaches deep tissues and/or the bloodstream, phagocytic cells play a pivotal role in the host defense against these invading pathogens during infection (Fang et al., 2017). Phagocytosis and intracellular survival assays were performed in RAW264.7 cells to assess the role of SsRex in the context of *S. suis*–phagocyte interactions. As shown in Figure 3B, there were no significant differences in the phagocytosis rates among SS2-1, Δ*rex*, and *C*Δ*rex* at timepoint of 0 h. However, significant differences were observed in the intracellular survival of SS2-1, Δ*rex*, and CΔ*rex* when they were co-incubated with macrophages for another 10 h (Figure 3B). Our results show that the mutant strain Δ*rex* exhibited significantly reduced resistance to killing after ingestion by macrophages in a time-dependent manner (Figure 3B), indicating that SsRex significantly contributes to bacterial survival in macrophages and that its deletion results in the attenuated virulence of SS2.

### Deletion of SsRex attenuates SS2 virulence in mice

Previous studies have indicated that Rex proteins are important for the virulence of bacterial pathogens (Bitoun et al., 2012; Bitoun and Wen, 2016). To determine whether SsRex is involved in the virulence of SS2, we compared the LD_50_ values of SS2-1, its mutant Δ*rex*, and the complementation strain CΔ*rex* in BALB/c mice. As shown in Table 3, at a high bacterial dose (4.20 × 10^7^ CFU per animal), mice in the WT and mutant groups all presented severe clinical symptoms associated with septicemia during the first 36 hpi, including depression, swollen eyes, weakness, and prostration. The death rate was 100% (8/8) in the WT group and 87.5% (7/8) in both the Δ*rex* and CΔ*rex* groups in the high dose trials. At an intermediate bacterial dose (8.40 × 10^6^ CFU per animal), most mice in the WT group also presented severe clinical symptoms and prostration during the first 36 hpi, and seven mice (87.5%) died from septicemia. Mice in the Δ*rex* group presented moderate clinical symptoms, and only one mouse (12.5%) died; four mice (50%) died from septicemia in the CΔ*rex* group. At a low bacterial dose (1.68 × 10^6^ CFU per animal), three mice died in the WT group. In contrast, no clinical symptoms were observed in the Δ*rex* and CΔ*rex* groups, and all the mice in these two groups survived until the end of the infection experiments. The LD_50_ value was 1.81 × 10^7^ CFU/mouse for the Δ*rex* group, which is 7-fold higher than that of the WT SS2-1 group (2.51 × 10^6^ CFU/mouse), the LD_50_ value was 8.40 × 10^6^ CFU/mouse for the CΔ*rex* group. The results suggest that Rex deficiency impaired the virulence of SS2 in a BALB/c mouse infection model.

**Table 3:** Values of LD_50_ on SS2-1, mutant and complemented strains for BALB/c mice.

**Strains**	**Dose of challenge (CFU)**	**Number dead /total**	**LD_50_(CFU/mouse)**
SS2-1	4.20 × 10^7^	8/8	2.51 × 10^6^
	8.40 × 10^6^	7/8	
	1.68 × 10^6^	3/8	
Δ*rex*	4.20 × 10^7^	7/8	1.81 × 10^7^
	8.40 × 10^6^	1/8	
	1.68 × 10^6^	0/8	
*C*Δ*rex*	4.20 × 10^7^	7/8	8.40 × 10^6^
	8.40 × 10^6^	4/8	
	1.68 × 10^6^	0/8	

### Differentially expressed proteins in the Δ*rex* mutant strain

**Figure 4:**
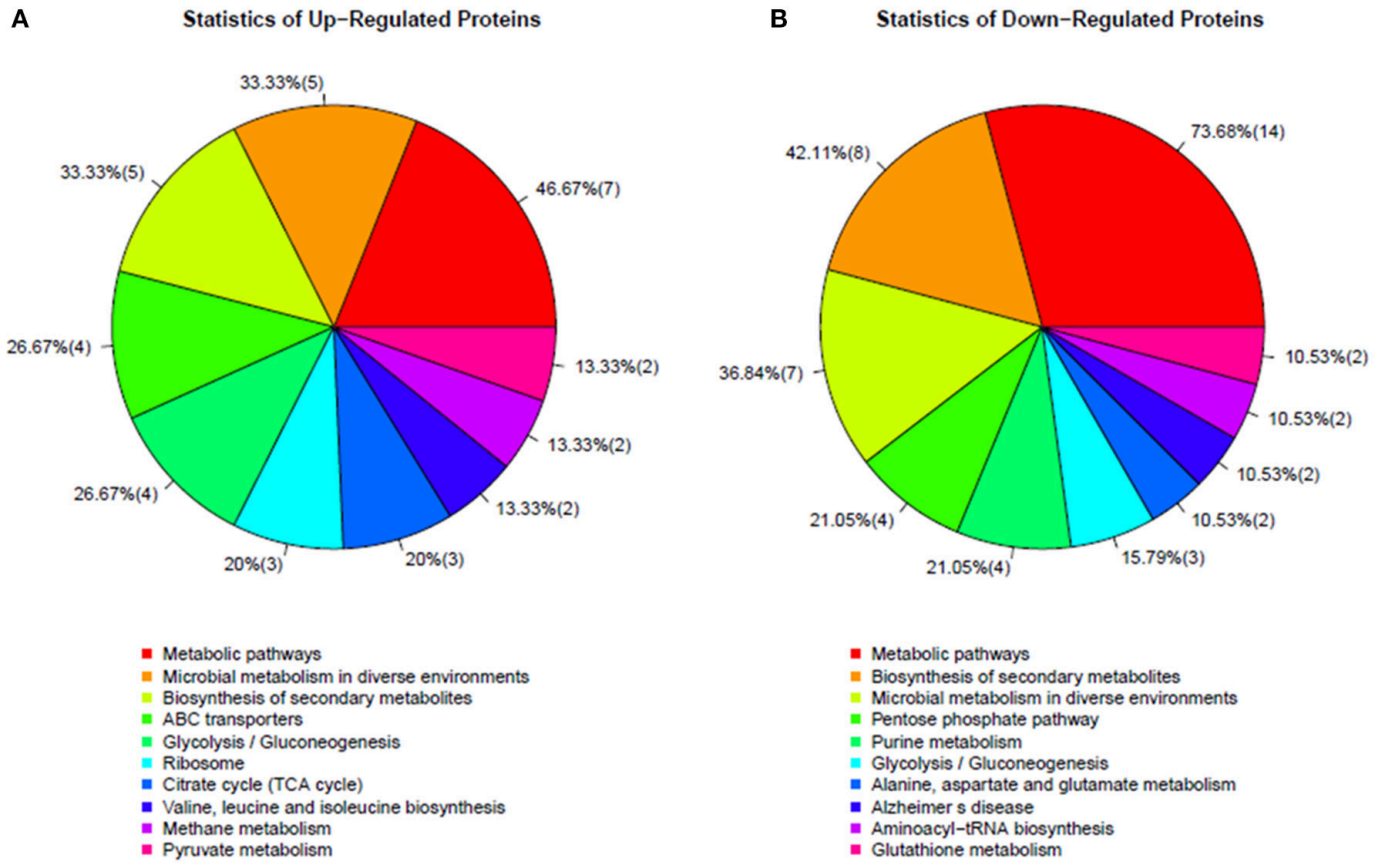
KEGG pathways enrichments in altered proteins in mutant strain Δ*rex*. **(A)** Up-regulated proteins and **(B)** Down-regulated proteins.

**Figure 5:**
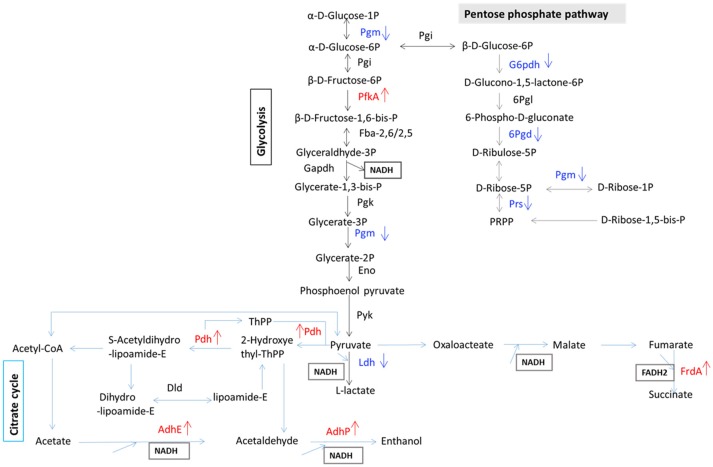
Schematic representation of SS2 metabolic pathways differentially regulated in carbohydrate metabolism. Differentially expressed proteins are involved in glycolysis, citrate cycle, and pentose phosphate pathway. Red color, up-regulated proteins; blue color, down-regulated proteins.

To determine which proteins are regulated by Rex, an iTRAQ analysis characterizing the differences in protein expression between the WT and Δ*rex* strains was performed. Compared with the WT strain SS2-1, 39 proteins were upregulated and 57 proteins were downregulated in Δ*rex*. The differentially expressed proteins (DEPs) between Δ*rex* and the WT strain are summarized in Supplementary Table 1. **(I) KEGG pathway analysis of DEPs**. To obtain an overview of the major perturbed functions in Δ*rex* compared with the WT SS2-1, a KEGG pathway enrichment analysis was conducted. Some of the DEPs in Δ*rex* were involved in metabolic pathways (7 proteins [46.67%] upregulated; 14 proteins [73.68%] downregulated), biosynthesis of secondary metabolites (5 proteins [33.33%] upregulated; 8 proteins [42.11%] downregulated), and microbial metabolism in diverse environments (5 proteins [33.33%] upregulated; 7 proteins [36.84%] downregulated) (Figure 4). Three metabolic pathways were significantly altered by *rex* deletion, including glycolysis/gluconeogenesis, the pentose phosphate pathway, and the citrate cycle (TCA cycle) (Figure 5), which were all associated with central metabolism (Richardson et al., 2015). Five proteins, including 6-phosphogluconate dehydrogenase (6Pgd), phosphomannomutase (Pgm), glucose-6-phosphate 1-dehydrogenase (G6pdh), L-lactate dehydrogenase (Ldh), and ribose-phosphate pyrophosphokinase (Prs), were significantly downregulated, and five proteins, including ATP-dependent 6-phosphofructokinase (PfkA), pyruvate/2-oxoglutarate dehydrogenase complex, dehydrogenase (E1) component (Pdh), fumarate reductase flavoprotein subunit (FrdA), and alcohol dehydrogenases (AdhE, AdhP) were significantly upregulated (Figure 5). **(II) SsRex regulates known virulence factors**. The SsRex deletion significantly reduced the virulence of SS2. Among the 32 virulence factors (VFs) predicted by the Virulence Factor Database (VFDB) (Chen et al., 2005), 14 known VFs of SS were among the DEPs in the Δ*rex* strain (Fittipaldi et al., 2012), including 9 downregulated proteins and 5 upregulated proteins, such as the transcriptional regulators TreR and Dpr, adenylosuccinate synthetase (PurA), autolysins (Ju et al., 2012), peptidase T (PepT) (Yu et al., 2016), type I restriction enzyme protein (HsdS) (Xu et al., 2017), aldehyde-alcohol dehydrogenase (AdhE) (Yu et al., 2018), foldase protein PrsA (Jiang et al., 2016), sialic acid synthase (NeuB), and arginine deiminase (ArcA) (Table 4). Another 18 VFs that have been reported in other pathogens were identified as DEPs in the Δ*rex* strain, including ATP-dependent zinc metalloprotease FtsH (Choi et al., 2015), metalloendopeptidas (PepO) (Agarwal et al., 2013, 2014), branched-chain-amino-acid aminotransferase (IlvE) (Santiago et al., 2012), adenylosuccinate lyase (PurB) (Connolly et al., 2017), and ATP-dependent protease ATP-binding subunit ClpL (Kwon et al., 2003) (Table 4, Supplementary Table 1).

**Table 4:** Virulence associated factors identified by iTRAQ in Δ*rex* and analyzed by qRT-PCR and EMSAs.

**Locus**	**Accession**	**Gene name**	**Description/Function**	**Fold change of protein expression (*Δrex*/WT)**	**Fold change of mRNA expression (*Δrex*/WT)**	**EMSA^a^**
SSU05_1076	A4VVA3	*ldh*	L-lactate dehydrogenase	0.44	0.47 ± 0.1	Shift
SSU05_1689	G7SLD4	*dpr*	Dps-like peroxide resistance protein	0.39	0.64 ± 0.13	Shift
SSU05_0766	A0A0Z8I809	*ilvE*	Branched-chain-amino-acid aminotransferase	0.27	0.63 ± 0.08	No shift
SSU05_0232	A0A0Z8CNM9	*treR*	Transcriptional regulator	0.59	0.45 ± 0.12	Shift
SSU05_1966	D5AK71	*purA*	Adenylosuccinate synthetase	0.39	0.67 ± 0.12	NT
SSU05_0039	A4VSC2	*purB*	Adenylosuccinate lyase	0.37	0.59 ± 0.03	NT
SSU05_1128	A4VVF5	*pepT*	Peptidase T	0.59	0.62 ± 0.11	NT
SSU05_0280	A0A0Z8JA13	*adhE*	Aldehyde-alcohol dehydrogenase	2.01	7.35 ± 0.37	Shift
SSU05_0624	A4VU01	*arcA*	Arginine deiminase	1.79	2.55 ± 0.15	Shift
SSU05_2153	A0A0H3MXX6	*frdA*	Fumarate reductase flavoprotein subunit	7.59	6.10 ± 0.34	Shift
SSU05_0279	A4VT08	*adhP*	Zn-dependent alcohol dehydrogenase	16.29	44.84 ± 2.2	NT
BM407_0341	A0A0Z8EN96	*clpL*	ATP-dependent protease ATP-binding subunit ClpL	2.29	1.32 ± 0.12	No shift

a *NT indicates that a gene was not tested by EMSA*.

### Transcriptional analysis

Alterations in the expression level of a protein may be due to a change in its mRNA level. Twelve genes selected based on the DEPs in the Δ*rex* strain, including seven downregulated genes and five upregulated genes, were measured via qRT-PCR in the mutant and WT strains. The *aroA* gene was used as a control housekeeping gene. As shown in Table 4, the transcript levels of the genes *dpr, ilvE, pepT,treR, purA, purB*, and *ldh* were significantly downregulated and those of *adhE, adhP, clpL, frdA*, and *arcA* were significantly upregulated in the mutant strain Δ*rex*, which matched well with the comparative proteomics data.

### DNA binding activity of SsRex

**Figure 6:**
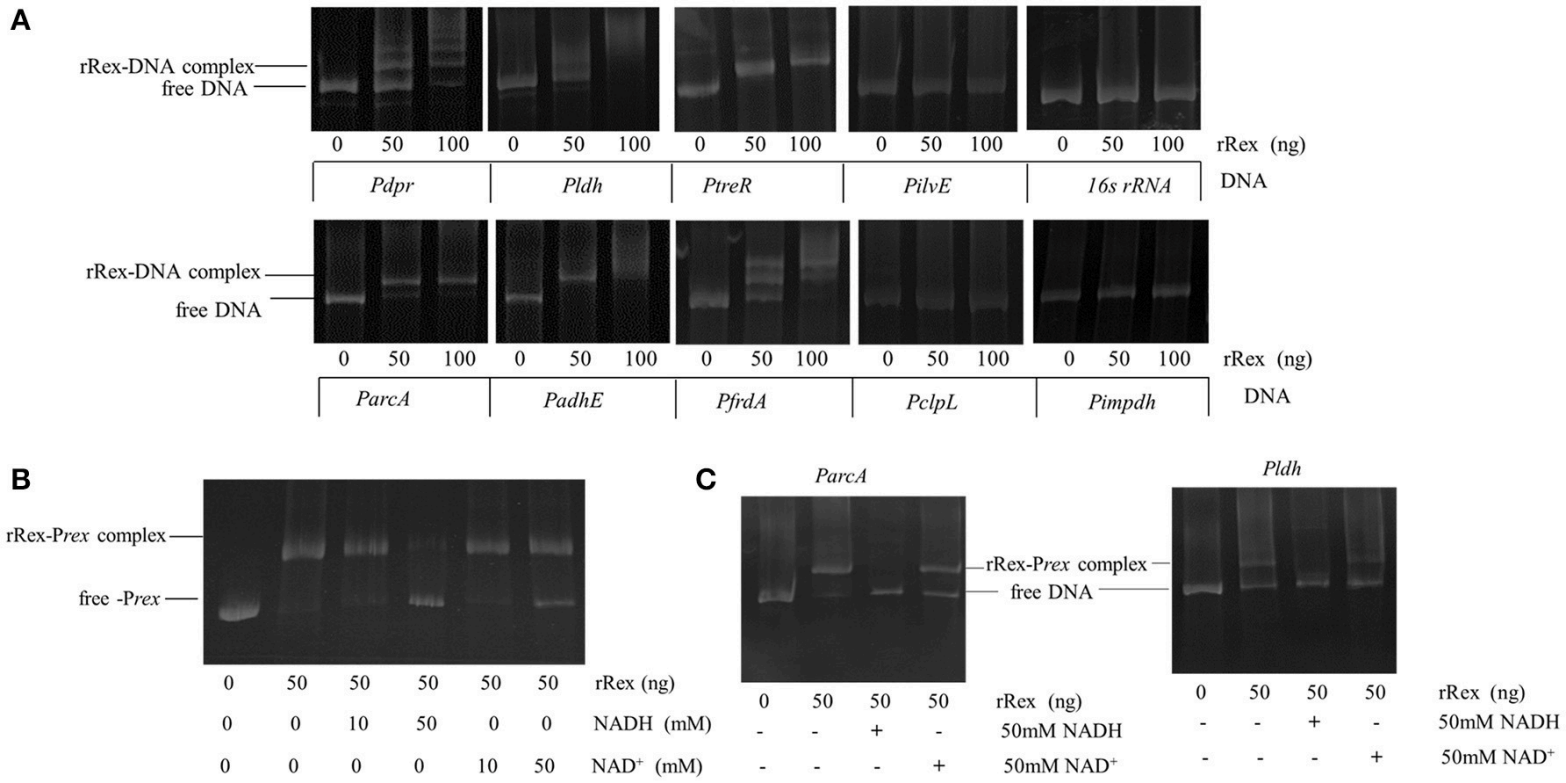
Determination of Rex binding abilities by EMSAs. **(A)** Binding of purified rRex to the promoter regions of different genes using EMSAs. The resulting protein–DNA complexes were separated from unbound DNA fragments using native polyacrylamide gels. The DNA fragments were visualized by ethidium bromide staining. Formation of stable Rex–DNA complexes resulted in one or more distinct shifted DNA bands. The promoter region of *impdh*, which is not regulated by Rex and whose promoter sequence lacks a putative Rex binding box, and 16sRNA DNA fragments were used as negative controls. **(B)** EMSAs were performed with PCR products of the promoter regions of *rex* incubated with 50 ng purified Rex protein and different concentrations of NAD^+^ and NADH. At lower concentration, NADH and NAD^+^ did not influence the rRex affinity to *Prex* and not change the mobility pattern. A higher concentration of NADH (50 mM) completely prevents the formation of rRex–*Prex* complexes, and 50 mM of NAD^+^ causes the complexes to dissociate slightly. **(C)** EMSAs were performed with PCR products of the promoter regions of *arcA* and *ldh* incubated with 50 ng purified rRex protein and 50 mM NAD^+^ or 50 mM NADH. The presence of 50 mM NADH inhibits promoter-DNA complex formation for *ParcA* and *Pldh*. However, the binding affinity between rRex and *ParcA* is slightly decreased in the presence of 50 mM NAD^+^, and rRex binds better to the *Pldh* when compared to *ParcA* under the same conditions.

Rex is an autoregulation repressor that binds to the operators of its target genes, modulating them in response to the cellular NADH/NAD^+^ level (Brekasis and Paget, 2003). To determine if SsRex is an authentic Rex transcriptional factor, we performed EMSAs to evaluate the binding ability of rRex to its promoter sequence (Rex-promoter, *Prex*). As shown in Figure 1B, recombinant Rex (rRex) can bind to DNA fragments containing *Prex*, in the same manner as that reported for *S. mutans* (Bitoun et al., 2012). To verify the direct role of Rex in the regulation of the altered genes, we used EMSAs to assess the interactions between rRex and the promoters of selected genes identified from the comparative proteome and transcriptional analyses. We tested the promoters of four upregulated genes (*adhE, arcA, clpL*, and *frdA*) and four downregulated genes (*dpr*,*ldh,ilvE*,and *treR*). Six promoters that contained known consensus Rex-binding motifs (shown in Figure 1C) displayed a dose-dependent mobility shift to higher molecular mass, indicating the formation of rRex–promoter complexes (Figure 6A). However, no mobility shift was observed when rRex was mixed with the promoter of *clpL and ilvE*, which demonstrated that the two genes were not directly regulated by Rex and whose promoter does not have an apparent Rex-box (Figure 6A). The results showed that *adhE,arcA, frdA, and ldh* were the Rex targets for SS2, similar to the previous reported in *S. aureus* (Pagels et al., 2010) and *S. mutans* (Bitoun et al., 2012; Bitoun and Wen, 2016). In addition, we found that the promoter regions of the two regulators (*dpr* and *treR*) also could be bound by rRex. To the best of our knowledge, these two new genes promoters were identified as the targets of Rex which has been reported for the first time. It was also worth noting that Rex may also act as a transcriptional activator, directly binding to the promoters of down-regulated genes, as well as a repressor which had been extensively reported in some bacteria (Brekasis and Paget, 2003; Gyan et al., 2006; Pagels et al., 2010).

To test if NADH and NAD^+^ affect the interaction between *S. suis* Rex and its cognate operators, EMSAs were performed using the target promoters *Prex, ParcA, and Pldh*. In the EMSA pre-reaction buffer, the rRex and the target promoters were pre-incubated for 20 min, and NADH and/or NAD^+^ (10 mM, and 50 mM, final concentration) were then added. As shown in Figure 6B, rSsRex can still bind to *Prex* with 10 mM of NAD^+^ or 10 mM of NADH in the EMSA reaction system, as evidenced by the mobility shift. The presence of 50 mM of NADH nearly completely prevents the formation of rRex–*Prex* complexes, whereas the addition of 50 mM of NAD^+^ did not result in a noticeable enhancement of Rex binding to the DNA fragment and exhibited a slight interference to the binding activity (Figure 6B). Additionally, rRex bound the *arcA* and *ldh* promoters under the similar conditions (Figure 6C). Although inclusion of lower concentration of NADH and NAD^+^ did not have a major effect on the mobility shift, the addition of higher concentration of NADH nearly completely abolished such binding activity (Figures 6B,C). It was unexpected that high concentration of NAD^+^ slightly impaired the formation of rRex-DNA complexes for some genes in *S. suis*. Similar findings had been reported for the Rex-family protein RSP in *Thermoanaerobacter ethanolicus* (Pei et al., 2011). The results show that SsRex could bind to its promoter region as an authentic Rex family protein and that the interaction was modulated by NADH and NAD^+^.

## Discussion

Bacterial pathogens use global regulatory networks to sense and modify gene expression in response to changing environments. In some Gram-positive bacteria, including *S. aureus* (Pagels et al., 2010), *B. subtilis* (Gyan et al., 2006; Wang et al., 2008), *E. faecalis* (Vesić and Kristich, 2013), and *S. mutans* (Bitoun et al., 2012; Bitoun and Wen, 2016), Rex proteins are global transcriptional regulators that play a pivotal role in the regulation of energy metabolism, stress tolerance, and virulence (Pagels et al., 2010; Bitoun et al., 2012; Vesić and Kristich, 2013; Laouami et al., 2014; Zhang et al., 2014; Hu et al., 2016; Liu et al., 2017). However, the role of Rex in SS2 had not been investigated in detail.

A bioinformatics analysis revealed that SsRex (possessing N-terminal DNA-binding sites and a C-terminal Rossmann-fold NAD(P)H/NAD(P)(+)-binding (NADB) domain) exhibits >78% amino acid sequence identity with the Rex homolog proteins in other *Streptococci*. This conserved structural feature ensures that the Rex protein can to bind specific DNA sequences to regulate the expression of target genes. In the present study, EMSAs revealed that both NADH and NAD^+^ could bind to rSsRex (Figures 6B,C). This finding suggests that SsRex is a typical member of the Rex family of regulatory proteins in that it has common structural features and similar regulatory mechanisms with other homolog proteins of the Rex family. Furthermore, our EMSA analysis of selected promoters (*adhE, arcA, frdA, dpr, ldh*, and *treR*) with known Rex-binding sites also demonstrated that these DNA sequences interact with rSsRex (Figure 6A) and the interaction was modulated by NADH and NAD^+^ (Figures 6B,C). Our additional analysis of these promoter regions/Rex-binding sites further showed that differences exist among various promoters in their nucleotide compositions and positions to the translation initiation site (ATG or GTG or TTG) (Figure 1C).Microarray data in *S. mutans* showed that Rex-deficiency effected transcription dissimilarly, suggesting either the involvement of other regulator(s) and/or regulation levels that depend on the nucleotide composition of the Rex-binding site (Bitoun et al., 2012). In *S. aureus*, Rex binding sites are localized within the promoter regions of its target genes or are only a few base pairs upstream or downstream of the promoter regions. This finding supports the idea that the binding of Rex at these sites prevents the binding of the RNA polymerase and hence hinders transcriptional initiation (Pagels et al., 2010). However, further studies are needed to elucidate the molecular mechanism of the Rex regulation involvement of SS2.

Rex proteins are important for the virulence of *S. mutans* (Bitoun et al., 2012; Bitoun and Wen, 2016). Here, the LD_50_ values of Δ*rex* calculated from BALB/c mouse infection model studies and *in vivo* colonization experiments (data not shown) show that the deletion of *rex* in *S. suis* resulted in altered bacterial pathogenicity and that *SsRex* facilitates the virulence of SS2. The attenuation of Δ*rex* may be due to a direct effect of *rex* on the expression of VFs. The comparative protein expression profiles showed that, among the 96 DEPs in Δ*rex*, there were 32 VFs predicted by the VFDB, including 20 downregulated and 12 upregulated proteins (Table 4, Supplementary Table 1) such as L-lactate dehydrogenase (Ldh), aminopeptidase T (AmpT), adenylosuccinate lyase (PurB), and metalloendopeptidase (PepO). In *Listeria monocytogenes*, AmpT is a member of the M29 family aminopeptidases that is involved in invasion and intracellular survival inside the host cells and is required for full virulence in a murine infection model (Cheng et al., 2015). In *S. aureus*, purine biosynthesis (PurA, PurB) is indispensable for its growth in human blood and *in vivo* pathogenicity in a zebrafish embryo model (Connolly et al., 2017). In *Streptococcus pneumoniae*, PepO is a multifunctional plasminogen- and fibronectin-binding protein that modulates the complement attack by binding to the complement component C1q (Agarwal et al., 2013, 2014).

Our iTRAQ analysis also identified 9 known *S. suis* VFs (Fittipaldi et al., 2012) among the 32 VFs with repressed expression in Δ*rex*, such as the transcriptional regulators TreR (Wilson et al., 2007) and Dpr (Zhang et al., 2012), PurA (Wilson et al., 2007), autolysins (Ju et al., 2012), PepT (Yu et al., 2016), and HsdS (Xu et al., 2017) (Table 4, Supplementary Table 1). In the SS2 virulent strain S735, *treR* and *purA* were identified as VFs by a novel signature-tagged mutagenesis system, and S735 with mutations in these genes had attenuated virulence in both mouse and piglet infection models (Wilson et al., 2007). In the SS2 virulent strain ZY05719, *hsdS* was reported to facilitate phagocytosis and survival in whole blood and to enhance the bacterial survival ability against a peroxidation environment (Xu et al., 2017). Furthermore, PepT was identified as a protein that is unique to a virulent strain, and its mutant strain Δ*pepT* had attenuated virulence in a zebrafish model (Yu et al., 2016). Therefore, downregulation of these known VFs may be associated with the decreased level of survival in whole blood and macrophages cells and the attenuated virulence of Δ*rex*.

In some bacteria, the metabolic pathways under Rex control are implicated in virulence (Richardson et al., 2015). The GO term and KEGG pathway analyses showed that several DEPs, such as 6Pgd, Pgm,G6pdh, Ldh, PfkA, Pdh, FrdA, and Adh, contribute to complicated cellular metabolic pathways (Figure 5), including glycolysis/gluconeogenesis, the pentose phosphate pathway, and the TCA cycle (Richardson et al., 2015). In the present study, the EMSAs showed that rRex can bind to the promoters of *ldh* and *frdA* (Figure 6A). Previous studies reported that LDH and ADH are also Rex targets (Larsson et al., 2005; Pagels et al., 2010; Mehmeti et al., 2011). In *S. aureus*, Rex is intimately involved in the survival of cells exposed to NO. Because NO inhibits the activities of terminal respiratory oxidases, nitrate reductase, and pyruvate formate lyase, it leaves LDH, a target of Rex, as the major means of regenerating NAD^+^ (Pagels et al., 2010). LDH enables the bacteria to resist host innate immunity, which means that modulating Rex function appropriately is a critical factor in the pathogenesis of *S. aureus* (Richardson et al., 2008). In *S. pneumoniae*, LDH, which is a key enzyme for pyruvate metabolism, was reported to be important for pneumococcal survival in blood, metabolism, and virulence (Gaspar et al., 2014). In SS2, 6PGD was found to be a good adhesin, and it induced protective immune responses in both mice and piglets (Tan et al., 2008, 2009).

During infection, phagocytic cells play a pivotal role against invading pathogens. The process of phagocytic killing begins with the engulfment of bacteria by endocytosis into phagosomes, which are then fused with lysosomes to form phagolysosomes (Chen et al., 2008). The formation of phagolysosomes, a hostile environment with various reactive oxygen species (ROS) and a lower pH, is essential for the intracellular destruction of pathogens. In our studies, Δ*rex* displayed defects in its ability to adapt to oxidative and acidic environmental conditions and had a decreased level of survival in macrophages. Similar results have been reported in other pathogens; for example, Rex-deficiency in *S. mutans* causes increased sensitivity to exogenous H_2_O_2_-mediated killing (Bitoun et al., 2012). In *E. faecalis*, a Rex-deficient mutant had an impaired ability to cope with oxidative stress (Mehmeti et al., 2011; Vesić and Kristich, 2013). Here, our iTRAQ results showed the downregulation of several proteins (such as Dpr, IlvE) that are involved in the stress response in Δ*rex*; these might be partly responsible for the phenotypes of defective growth under conditions of oxidative and acidic stresses. Dpr is especially crucial for the H_2_O_2_ resistance of SS2, and inactivation of *dpr* led to a nearly complete loss of the SS2 H_2_O_2_ defensive capability (Zhang et al., 2012). The EMSAs also revealed that rSsRex can bind to the promoters of *dpr* (Figure 6A), suggesting that Rex may directly regulate oxidative stress response of SS2. In *S. mutans*, IlvE was upregulated in a proteome analysis under acidic stress conditions, and a deletion of *ilvE* caused a decrease in acid tolerance (Santiago et al., 2012). In SS2, a group of global regulators such as Spx proteins (Zheng et al., 2014), SalK/SalR (Li et al., 2008), Ihk/Irr (Han et al., 2012) were positively related to oxidative stress tolerance of bacterial, these genes mutants exhibited impaired growth in the presence of H_2_O_2_ *in vitro* and significantly decreased survival *in vivo*. These findings suggest that the Rex regulator in SS2 might not only be responsible for the defective growth of the mutant strain under stress conditions, but also facilitate the survival of SS2 within the host.

In our study, we found that a Rex mutation made the SS2 more susceptible to acidic and oxidative environments and easily to be eliminated in whole blood. However, there was no significant difference between mutant strain Δ*rex* and complemented strain CΔ*rex* in the resistance to an H_2_O_2_ environment (for a 30-min incubation) and the whole blood environment. It is interesting that the complementation of Δ*rex* was able to restore the WT phenotype in the acid stress assay, but, it was not able to rescue the mutant phenotypes in the oxidative stress assay (for a 30-min incubation) or in the blood survival assay. Similar observations have been reported in other studies (Behlau and Miller, 1993; Ju et al., 2012; Mcgillivray et al., 2014; Lewis et al., 2015). The most likely reasons are that (i) Rex functional complementation is mediated by a plasmid, which might be different from complementing the target genes into the genome of bacteria (Dortet et al., 2011; Lewis et al., 2015). A previous study reported that the plasmid-mediated complementation for Rv2745c gene in *Mycobacterium tuberculosis* resulted in either a partial or, a delayed complementation of the phenotypes (Mcgillivray et al., 2014), and (ii) the transcription level of *rex* and/or that of some genes within Rex regulon of the complemented strain CΔ*rex* may occur within a few microenvironments (such as in whole blood), which might be due to a secondary response of genes repression. Further researches are needed to identify that the genes expression patterns under Rex regulation in whole blood and the regulatory elements within Rex regulation networks that impact expression of *rex* at different environmental conditions.

In summary, bioinformatic, mutational, and proteomic analyses as well as EMSAs were used to identify and characterize the Rex regulator of SS2. The present study has clearly demonstrated that SsRex is an authentic Rex transcriptional factor based on the following evidence: (i) it was able to bind to promoter fragments *in vitro*, indicating that, at least in some cases, the effect of Rex is mediated by direct interaction with promoters such as those for Rex, ArcA, and AdhE, and (ii) it impacted the cellular metabolism, oxidative stress tolerance, and virulence, as previously reported for Rex homolog proteins in other pathogens (Pagels et al., 2010; Bitoun et al., 2012; Laouami et al., 2014; Bitoun and Wen, 2016). We also demonstrated that the Rex mutant strain showed a reduced ability to colonize host tissues and a decreased survival in whole blood or following phagocytosis, which partially explains the attenuated bacterial pathogenicity of the Rex mutant strain in animal models. Further investigations of the Rex-related protein expression patterns under *in vivo* infection conditions or host environment mimics are needed; however, our study provides novel insights into the requirement for Rex in the pathogenesis of SS2 infection.

## Author contributions

HZ, YW, KH conceived and designed the experiments. HZ, YW, LH performed the experiments. HZ, YW, JZ analyzed the data. YN, ZY, DW, AM, HF contributed reagents materials analysis tools. HZ, YW, KH wrote the paper. All authors read, advised, and approved the final manuscript.

### Conflict of interest statement

The authors declare that the research was conducted in the absence of any commercial or financial relationships that could be construed as a potential conflict of interest.
