# Supplementary material for: The Redox-Sensing Regulator Rex Contributes to the Virulence and Oxidative Stress Response of Streptococcus suis Serotype 2
Source: Front Cell Infect Microbiol. 2018 Sep 18;8:317. doi: 10.3389/fcimb.2018.00317 (PMC6154617; doi:10.3389/fcimb.2018.00317)
Supplement: Supplementary file 1 [file Table_1.docx]

Table S1 List of differentially expressed proteins in *Δrex*

| Accession | Description/Function^a^ | Fold change |
| --- | --- | --- |
| A4VSA0 | **ATP-dependent zinc metalloprotease FtsH** | 0.3250873 |
| A4VY59 | **Predicted metalloendopeptidase,PepO** | 0.3467368 |
| D5AK71 | **Adenylosuccinate synthetase ,PurA** | 0.3872576 |
| A4VSC2 | **Adenylosuccinate lyase ,PurB** | 0.3698282 |
| A4VU63 | **Phosphomannomutase,Pgm** | 0.2910717 |
| D5AJK3 | **6-phosphogluconate dehydrogenase ,6Pgd** | 0.5807644 |
| A0A0Z8XZK6 | **Tellurite resistance protein, TelA** | 0.1887991 |
| A4VVA3 | **L-lactate dehydrogenase, Ldh** | 0.4446313 |
| A4VXS8 | **Aminopeptidase T, AmpT** | 0.5754398 |
| A4VVF5 | **Peptidase T,PepT** | 0.5861381 |
| A4VVX1 | **N-acetylmuramoyl-L-alanine amidase /autolysins** | 0.5807644 |
| G7SLD4 | **Dps-like peroxide resistance protein ,Dpr** | 0.3872576 |
| A0A0Z8CNM9 | **Transcriptional regulator treR** | 0.5861381 |
| A0A0Z8I809 | **Branched-chain-amino-acid aminotransferase, IlvE** | 0.2654606 |
| D5AIS8 | **Type I restriction enzyme EcoEI M protein,HsdS** | 0.3280953 |
| D5AIW8 | **Tetratricopeptide repeat family protein** | 0.4698941 |
| A4VTN3 | **Collagenase and related protease** | 0.4487454 |
| A4VTN2 | **Collagenase and related protease** | 0.3467368 |
| A4VWT4 | **Phosphomannose isomerase ,Man1** | 0.4092606 |
| A4VVJ9 | **ATP synthase subunit beta ,AtpD** | 0.452897579 |
| A4VTY0 | SAM-dependent methyltransferase related to tRNA (Uracil-5-)-methyltransferase | 0.3499452 |
| A4VVL4 | Signal recognition particle receptor FtsY | 0.3981072 |
| G7SHU1 | ABC transporter ATPase | 0.6546362 |
| A4VWI7 | DNA gyrase subunit B | 0.613762 |
| A4VUA8 | DNA helicase | 0.6426877 |
| A4VUK7 | ATPase component of ABC transporters with duplicated ATPase domains | 0.5970353 |
| A4VY90 | ATPase related to the helicase subunit of the Holliday junction resolvase | 0.510505 |
| A4VXV2 | ATPases with chaperone activity, ATP-binding subunit | 0.5445026 |
| U5UHD9 | Lipopolysaccharide biosynthesis protein | 0.5861381 |
| A0A123TNK0 | GTPase | 0.4130475 |
| A4VUC8 | GTPase Obg | 0.3076097 |
| A4VW74 | GTPase Era | 0.3372872 |
| A0A0Z8ILK3 | Aminotransferase patA | 0.5058246 |
| A4VV92 | Ribose-phosphate pyrophosphokinase | 0.2421029 |
| A4VWM9 | Uracil phosphoribosyltransferase | 0.2376841 |
| A0A0Z8FED6 | Glucose-6-phosphate 1-dehydrogenase, G6pd | 0.4570881 |
| A4VU69 | Pyruvate/2-oxoglutarate dehydrogenase complex, dihydrolipoamide dehydrogenase (E3) component, and related enzymes | 0.4055085 |
| A4VX00 | NH(3)-dependent NAD(+) synthetase ,NadE | 0.2376841 |
| A4VVT7 | UPF0342 protein SSU05_1260 | 0.2228435 |
| D5AG24 | Deoxyguanosinetriphosphate triphosphohydrolase-related protein | 0.4246195 |
| D5AIR2 | UDP-glucose 4-epimerase | 0.4920395 |
| A4VX47 | UDP-N-acetylmuramate--L-alanine ligase, murC | 0.310456 |
| A4VVU8 | Cytidylate kinase | 0.3698282 |
| A4VVU0 | 3-dehydroquinate dehydratase, aroD | 0.4742419 |
| D5AHG1 | Probable GTP-binding protein EngB | 0.2910717 |
| A4VTR0 | HD superfamily phosphohydrolase | 0.6486344 |
| A4VWC2 | Purine nucleoside phosphorylase DeoD-type | 0.4830588 |
| A4VWB3 | Predicted sugar phosphatases of the HAD superfamily | 0.2488858 |
| A4VVH5 | Uncharacterized protein | 0.608135 |
| A4VV20 | Uncharacterized protein | 0.3311311 |
| A4VTN7 | Uncharacterized protein | 0.519996 |
| U5UDP7 | Uncharacterized protein | 0.285759 |
| A4VTP6 | Lysine--tRNA ligase | 0.2754229 |
| A4VVH7 | Phenylalanine--tRNA ligase beta subunit | 0.6546362 |
| A4VSM8 | 10 kDa chaperonin | 0.2558585 |
| A0A0Z8JA13 | **Aldehyde-alcohol dehydrogenase ,AdhE** | 2.0137245 |
| A4VT08 | **Zn-dependent alcohol dehydrogenase ,AdhP** | 16.292961 |
| D5AIQ2 | **Cation transporting ATPase** | 1.7060826 |
| D5AIK9 | **Surface antigen SP1** | 1.8365383 |
| A0A0H3MXU4 | **Anaerobic ribonucleoside-triphosphate reductase ,NrdD** | 2.1281393 |
| A0A0Z8EN96 | **ATP-dependent protease ATP-binding subunit ClpL** | 2.2908674 |
| A0A0H3MXX6 | **Putative fumarate reductase flavoprotein subunit, FrdA** | 7.5857755 |
| G7SNC3 | **Peptide methionine sulfoxide reductase,MrsA** | 3.7670384 |
| A4VVR5 | **Foldase protein, PrsA** | 3.0478952 |
| A4VTV5 | **Sialic acid synthase** | 2.2284349 |
| A4VU01 | **Arginine deiminase ,ArcA** | 1.7864878 |
| A4VY63 | **High-affinity zinc uptake system protein, ZnuA** | 1.8197009 |
| D5AJI8 | Predicted periplasmic solute-binding protein | 1.9588448 |
| A4VXG8 | Pyruvate/2-oxoglutarate dehydrogenase complex, dehydrogenase (E1) component, eukaryotic type, alpha subunit ,Pdh | 1.5995574 |
| A4VXG7 | Pyruvate/2-oxoglutarate dehydrogenase complex, dehydrogenase (E1) component, eukaryotic type, beta subunit ,Pdh | 1.6749423 |
| A4VTS0 | ATP-dependent 6-phosphofructokinase | 2.1478307 |
| A4VWU4 | Methionine aminopeptidase | 2.2080046 |
| A4VSN2 | 30S ribosomal protein S7 | 1.9408863 |
| A4VSG7 | 30S ribosomal protein S8 | 1.9952618 |
| A4VSN1 | 30S ribosomal protein S12 | 2.884032 |
| A4VXF9 | 30S ribosomal protein S18 | 2.4434308 |
| A4VVB6 | 30S ribosomal protein S20 | 1.7701088 |
| A4VSF9 | 50S ribosomal protein L22 | 2.2908674 |
| A4VSG4 | 50S ribosomal protein L14 | 2.2908674 |
| U5UFQ4 | 50S ribosomal protein L18 | 3.8370726 |
| G5L2B4 | ABC-type multidrug transport system, ATPase and permease component | 2.7289771 |
| A4VW38 | ABC-type polar amino acid transport system, ATPase component | 1.9230917 |
| D5AHC4 | Putative ABC transporter, ATP-binding protein | 1.5135614 |
| A4VW82 | Putative ABC transporter | 2.8575905 |
| A4VU44 | Phosphoglycerate dehydrogenase and related dehydrogenase | 3.0199512 |
| A0A123SYN8 | Phosphotransferase family protein | 1.5416998 |
| A4VX81 | Major membrane immunogen, membrane-anchored lipoprotein | 2.2080046 |
| A4VTS3 | Glycine/D-amino acid oxidases (Deaminating) | 5.19996 |
| A4VUU8 | Translation elongation factor (GTPases) | 2.5351284 |
| A4VXH3 | Translation initiation factor IF-2 | 1.7218683 |
| A0A0K2E4Y0 | Translation initiation factor IF-3 | 1.6443714 |
| A4VTD4 | UPF0176 protein SSU05_0406 | 1.8535311 |
| A4VXA0 | Glutamine amidotransferase, class I | 1.8197009 |
| G7SK61 | Putative glutamine ABC transporter, glutamine-binding protein/permease protein | 2.1877613 |

a:Virulence associated factors identified by VFDB are shown in bold.
