# Supplementary material for: The Redox-Sensing Regulator Rex Contributes to the Virulence and Oxidative Stress Response of Streptococcus suis Serotype 2
Source: Front Cell Infect Microbiol. 2018 Sep 18;8:317. doi: 10.3389/fcimb.2018.00317 (PMC6154617; doi:10.3389/fcimb.2018.00317)
Supplement: Supplementary file 2 [file Data_Sheet_1.docx]

**Figure legends**

Figure S1. Transcriptional analysis of the *rex* operon in SS2-1. Reverse transcription-PCR (RT-PCR) analysis of *rex* operon with genomic DNA (gDNA), cDNA and cDNA-RT (cDNA reaction mixtures without reverse transcriptase) as templates. Lanes 1-3 represent the amplification using primers CT-P1 / CT-P2. Lanes 4-6 represent the amplification using primers CT-P3 / CT-P4.

Figure S2. Construction and characterization of the isogenic *rex* mutant *△rex.*

**(A)**Schematic representation of the chromosomal structures before (up) and after (down) double-crossover recombination events between pSET4s-△Rex and the chromosome of SS2-1. The chloramphenicol resistance cassette (*Cat*) is represented by blue boxes. Open arrows and boxes indicate the *rex* gene. The *spc* gene and Ts replication origin are represented by orange and red arrows, respectively. The thick bars represent the flanking regions of the *rex* gene.

**(B)** Multiple-PCR confirmation of knockout mutant strain *△rex.* Lane 1: primers CPS2J1/CPS2J2, SS2-1, Lane 2: primers CPS2J1/CPS2J2, *△rex*, Lane 3: primers IN1/IN2, SS2-1, Lane 4: primers IN1/IN2, *△rex*, Lane 5: primers CAT1/CAT2, SS2-1, Lane 6: primers CAT1/CAT2, *△rex*, Lane 7: primers CAT1/OUT2, SS2-1, Lane 8: primers CAT1/OUT2, *△rex*, Lane 9: primers CAT2/OUT1, SS2-1, Lane 10: primers CAT1/OUT2, *△rex*, M1: Marker DL2000, M2: Marker DL5000.

**(C)** Multiple-PCR confirmation of complemented strain C*△rex*. Lane 1: primers IN1/IN2, SS2-1, Lane 2: primers IN1/IN2, *△rex*, Lane 3: primers IN1/IN2, C*△rex*, Lane 4: primers CAT1/CAT2,SS2-1, Lane 5: primers CAT1/CAT2, *△rex*, Lane 6: primers CAT1/CAT2, C*△rex*, Lane 7: primers Spc1/Spc2, SS2-1, Lane 8: primers Spc1/Spc2, *△rex*, Lane 9: primers Spc12/Spc2, *C△rex*, M: Marker DL2000.

**(D)** qRT-PCR ananlysis of the *rex* expression in SS2 WT and mutant strains.

Total RNAs were extracted at the log phase of SS2 strains at an OD600 value of 0.6-0.8 and used for qRT-PCR. The mRNA levels of *rex* in the different SS2 strains were normalized to that of aroA. The expression level value of *rex* in the WT strain SS2-1 was set as 1.0.Results were shown as relative expression ratios compared to SS2-1. The qRT-PCR showed that the mRNA levels of *rex* in Δ*rex* , CΔ*rex* with antibiotic, and CΔ*rex* in the absence of antibiotic pressure were decreased by 1.0, 0.32, and 0.45, respectively, compared with the WT. The results confirmed the *rex* was transcribed after complementation regardless of the antibiotic pressure.

Figure S3. Eﬀect of the SsRex on the adherence and invasion ability of SS2. Evaluating the eﬀects of Rex mutation on adherence and invasion ability of SS2. The 10^5^ HEp-2 cells maintained in 24-well plate were infected with SS2 (10^6^ CFU) at a MOI :10 and incubated at 37°C for 2 h. The infected cells were lysised by sterile distilled water followed by plating the lysis on THA for cultivation in the 37 C incubators for 24 hours, determining the CFU numbers of bacteria adhered on HEp-2 cells in indicated groups. The adherence experiments were repeated at least three times (A). The SS2 (10^6^ CFU) at a MOI:10 was incubated with 10^5^ HEp-2 cells at 37°C for 2 h. The infected cells were washed 3 times with PBS before being kept in DMEM culture medium containing gentamicin (100μg/ml) and penicillin (5μg/ml) for 2 h to kill the bacteria outside the HEp-2 cells. Then the HEp-2 cells were washed thrice by PBS followed by calculating the CFU by plating the lysis of the cells for thrice in indicated groups. The percentage of the CFU was normalized to WT group designed as 100%.
