# Supplementary figures and images for: The Redox-Sensing Regulator Rex Contributes to the Virulence and Oxidative Stress Response of Streptococcus suis Serotype 2
Source: Front Cell Infect Microbiol. 2018 Sep 18;8:317. doi: 10.3389/fcimb.2018.00317 (PMC6154617; doi:10.3389/fcimb.2018.00317)

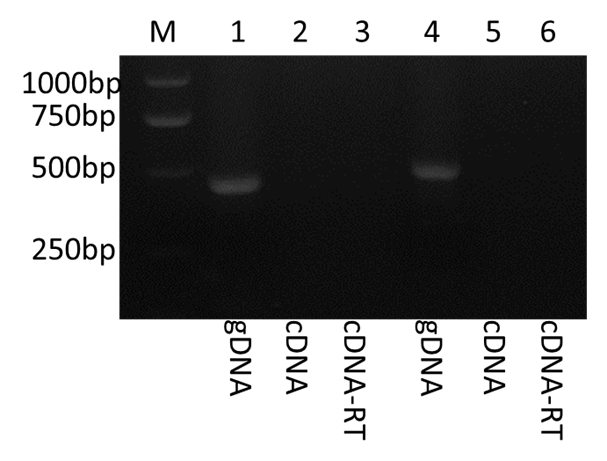

Supplement: Supplementary file 3 [file Image_1.TIF]

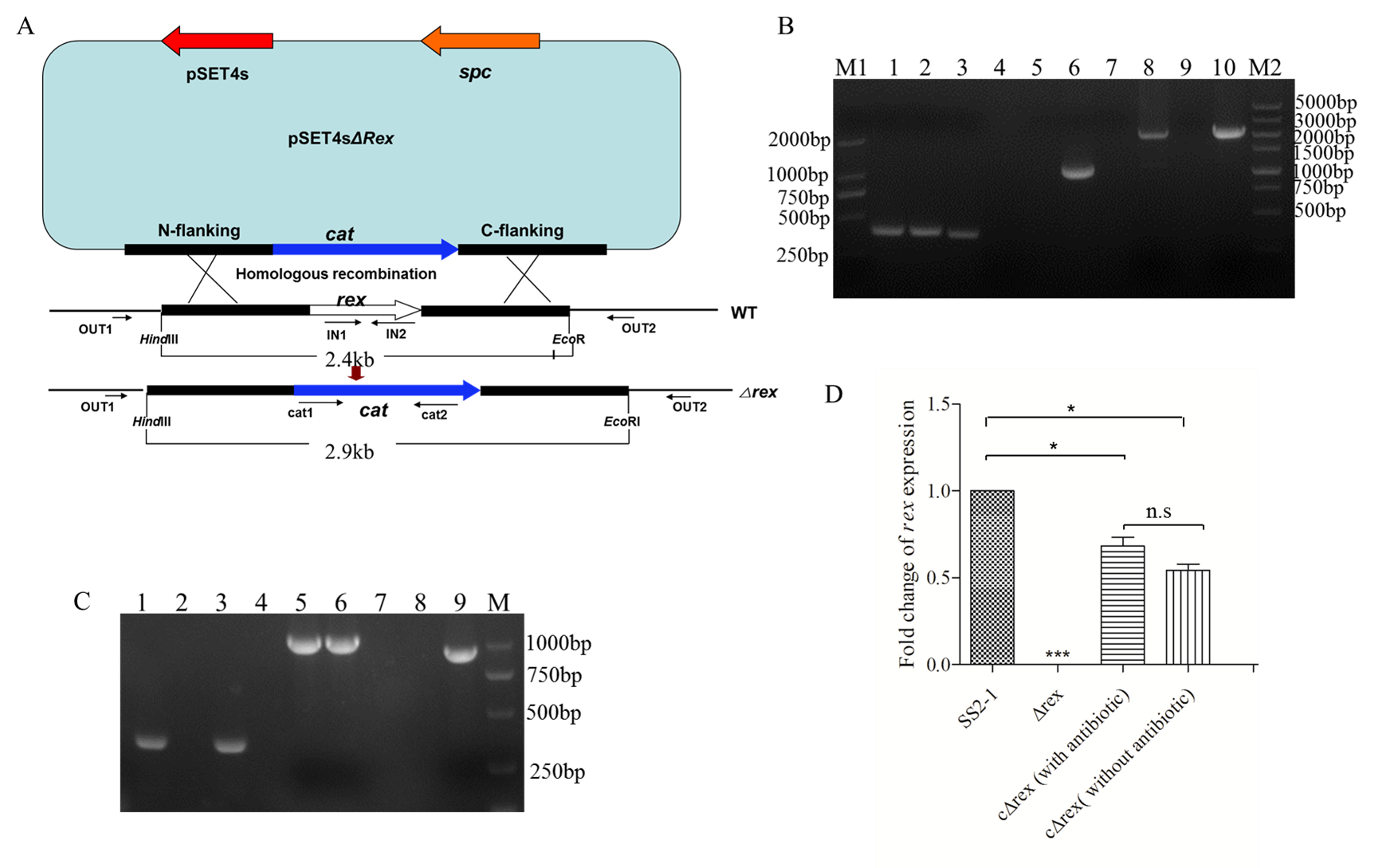

Supplement: Supplementary file 4 [file Image_2.TIF]

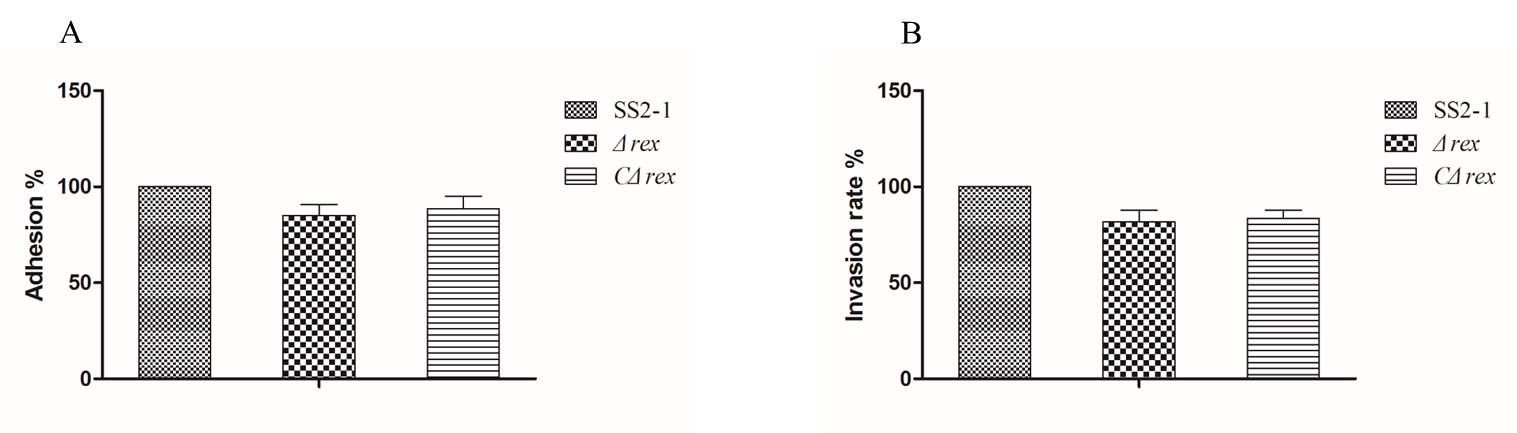

Supplement: Supplementary file 5 [file Image_3.TIF]
